# Supplementary material for: Outpatient Readmission in Rheumatology: A Machine Learning Predictive Model of Patient’s Return to the Clinic
Source: J Clin Med. 2019 Aug 2;8(8):1156. doi: 10.3390/jcm8081156 (PMC6723392; doi:10.3390/jcm8081156)
Supplement: Supplementary file 1 [file jcm-08-01156-s001.zip › Supplementary_File_Sections_R2.docx]

**SUPPLEMENTARY FILE**

**Outpatient Readmission in Rheumatology: A Machine Learning Predictive Model of Patient’s Return to the Clinic**

Authors:

Alfredo Madrid-García, Judit Font-Urgelles, Mario Vega-Barbas, Leticia León-Mateos, Dalifer Freites, Cristina Lajas, Esperanza Pato, Juan Angel Jover, Benjamín Fernández-Gutiérrez, Lydia Abásolo-Alcazar, Luis Rodríguez-Rodríguez.

**Database Clean-up**

From April 1^st^, 2007 to November 30^th^, 2017, information from 87,719 patients was stored in Medi-LOG, including both prevalent and incident patients. In order to ensure data integrity, we followed consecutive steps assessing the quality of the data (See **Supplementary Figure S1**).

1. Duplicated patients: We detected 231 patients with two different ID numbers and 1 patient with three different ID numbers. Hence, information regarding the same patient was merged into a unique ID number. The last ID number used was the one kept and the others (n = 233) were removed.
2. Lack of visit information: we observed 2,882 patients with only demographic and administrative data lacking information in the rest of the tables. In addition, two more patients were excluded due to an invalid year visit date (2026 and 2045)
3. Dummy patients: Patients used for testing the database (n = 2) were also removed.

After the previous steps a total number of 84,600 patients remained. Next, the following steps were carried out based on data coherence among tables.

1. Data coherence: Patients’ registers were matched and compared among the different tables of the database to avoid possible discrepancies. Natural Language Processing (NLP) of the data stored as free text in the visit information table was carried out in order to extract information regarding diagnoses, treatments, health status and care plans and match it to the existent codified information in other tables. Only patients with coherence regarding diagnoses, treatments and health status in all their visits were not removed.
   1. Based on this matching process, we decided to retain only patients seen for the first time from April 1^st^, 2007 onwards, as previous registers showed a high degree of inconsistency.
   2. Moreover, for those visits after April 2007, diagnoses information was considered to be valid if all diagnoses given or confirmed in each visit matched between the visit information table and the diagnoses table.
   3. Treatments information was considered to be valid if all treatments given or confirmed in each visit matched between the visit information table and the treatment table. Registers with no given treatment in both tables were also considered valid.
   4. The health status information was considered to be valid if the information collected in each visit matched between the visit information table and the health status table.

After the data coherence step 35,595 patients remained.

1. In the last step, patients with missing demographic data were excluded, as well as a patient with an invalid health status value*.*

After the previous quality control steps, 35,586 patients were included in the HCSC–MSK cohort.

**Predictors**

The predictors included in our analyses were:

*Demographic-related (3):* gender, age at first visit in the rheumatology outpatient clinic, and age at discharge.

*Diagnosis-related (121):* the presence/absence of the diagnoses included in the **Supplementary Excel File Categories**, **Diagnoses 2^nd^ categorization Sheet** was assessed at first visit in the rheumatology outpatient clinic, at the first visit of each episode (defined as the follow-up time between the first visit in our outpatient clinic/a first visit after a previous discharge, and the visit when the patient is discharged), at discharge, and in any visit within the 90 and 182 days before discharge (considering the current discharge visit). In addition, the number of diagnosis given at discharge was also analyzed.

*Treatment-related (81)*: the presence/absence of the prescription included in the **Supplementary Excel File Categories**, **Treatments 2nd categorization Sheet** was assessed at first visit in the rheumatology outpatient clinic, at the first visit of each episode, at discharge, and in any visit in the 90 and 182 days before discharge. In addition, the number of drugs prescribed at discharge was also analyzed.

*Quality of life-related (33)*: it was assessed using the Rosser Classification Index[1], and both subscales of distress/pain and disability, at first visit in the rheumatology outpatient clinic, at the first visit of each episode, at discharge, and the mean and median value of all visits in the 90 and 182 days before discharge (both including and excluded the values of the discharge visit).

*Other clinical-related variables (10)*: including the number of visits until discharge since the first visit in our clinic; the number of visits until discharge since the last discharge; the number of previous discharges; the follow-up duration until discharge from the first visit in the rheumatology outpatient clinic; the follow-up duration until discharge from the first visit following a previous discharge (episode); the follow-up duration until discharge from the previous visit regardless it was or not a discharge; the follow-up duration until discharge from the previous discharge; and patient’s occupation (active workers, housework, retired, or student) at first visit, at discharge and at the first visit of each episode.

*Comorbidity-related (155)*: the presence/absence of the comorbidities and concomitant treatments included in the **Supplementary Excel File Categories**, **Final Comorbidities Categories Sheet** was assessed at discharge.

**Sensitivity and specificity of the discharge status variable**

We assessed the error in the codification of the “discharge status” variable. To that aim, we carried out a manual review (JFU; rheumatologist) of the clinical notes corresponding to randomly selected visits classified by the rheumatologists as “discharge” or “not discharge”, in order to determine whether the recorded discharge status during the visit agreed with the clinical notes. Before the selection, we stablished different strata, based on factors which may affect the quality of the codification, to ensure that each subgroup was adequately represented:

- Year of the visit: To consider possible changes in the clinical practice procedure over time. Eleven years, from 2007 to 2017 were considered for the classification.
- Whether a patient was diagnosed with a chronic inflammatory disease in that visit.
- Time interval (in months) in which a patient returned to the clinic. Five different intervals were specified (no return, 0-3 months, 3-6 months, 6-12 months, >12 months).

Based on the combination of these factors, we stablished up to 110 strata, from which we randomly selected up to 22 visits classified as “discharge”, and up to 22 visits classified as “not discharge”. After carrying out the random selection of visits, we observed that for each strata, we could select between 1 and 22 visits classified as “discharge” (mean number of visits per strata = 19; 2,055 in total) and between 2 and 20 visits classified as “not discharge” visits (mean number of visits per strata = 19; 2,041 in total). Sensitivity and specificity values of the *discharge status* variable were calculated based on the codified values of that variable and the assessment of the care plan from the clinical notes review (**Supplementary Table S3**). Sensitivity and specificity was estimated for a) all review visits, b) only for those with no return to the clinic, c) only for those returning to the clinic, d) only for those returning to the clinic in the first 12 months after discharge, and e) and only for those returning to the clinic in the first 12 months after discharge excluding the first one month, two months, etc. This approach was carried out to assess the possibility that some visits codified by the rheumatologist as “discharge” and followed by an early return to the clinic were in fact “false discharges”, meaning the physician wrongly selected “discharge” as the care plan for the patient but in reality, the patient was scheduled to return.

**Model Fine-Tuning**

Two different feature selection techniques were considered for the model’s development. The first one was based on Random Forest variable importance report, from which we estimated using an internal measure of variable importance (VIMP), described below. The second one, and in order to reduce the high dimensionality of the predictors, was based on principal component analysis (PCA).

The tuning parameters were the number of trees included in the forest (ntree), the number of randomly selected variables used in each node to split the tree (mtry), the number of cases from the training dataset artificially increased by using the Synthetic Minority Over-Sampling Technique (SMOTE)[2], the relationship between the number of instances included as cases and controls when growing the trees in the algorithm (sampsize) and finally the number of predictors included in the model, based either on their predictive capacity (using an internal measure of variable importance or VIMP) or in the cumulative proportion (CP) of variance using Principal Component Analysis. PCA feature reduction was performed after applying SMOTE technique[3,4].

Regarding the number of trees, four values were arbitrary chosen: 100, 250, 500 (the default value) and 1000.

Regarding mtry, values were selected using as the central value the default mtry, which is the squared root of the total number of predictors (*P*) included in the model. Two higher and lower values were also used (default ±5 and default ±10). When the default number was lower than 10 or 5, the values “default – 10” and “default – 5” were not included, as mtry cannot take negative or zero values.

Regarding, the number of cases, it is important to highlight that our dataset was unbalanced, meaning that the number of discharges followed by outpatient readmission (15.3%, 9.6%, and 9.5% in the training, validation and test datasets respectively) was outnumbered by the non-readmission discharges. To deal with this issue, the number of cases in the training dataset was artificially increased by using SMOTE. With this technique, new datasets were created by resampling the original training set. Hence, two parameters were defined: the k-nearest neighborhood of each case to be used to create new instances (k), as well as the number of new instances of the minority class to be created (perc*.*over*).* Regarding the majority class, the parameter perc.under was modified according to the *perc.over* so the number of controls was the same in all the models.

For *k,* six different values were considered: 1, 2, 3, 5, 7, 9; for perc.over, the number of cases in the training dataset was increased by 100%, 200%, 300%, and 400%. On the other hand, perc.under*,* which depends on the perc.over value, defines the number of instances of the majority class to be used. Therefore perc.under, values were calculated as follows so the number of controls was the same in all models:

$$perc.under$$

$$=$$

$$\frac{\left( \left( Multiplier of minority class \right)+ Minority class instances \right)*100}{Multiplier of minority class}$$

Where:

$$Multiplier of minority class=\frac{perc.over}{100}* Minority class instances$$

Therefore, for the values of perc.over of 100 to 400, perc.under took the values 554.11, 277.05, 184,70, and 138.53 respectively (see **Supplementary Table S2**)**.** With this combination of perc.over and perc.under values, we assured that the number of instances of the majority class was fixed to 10,988, while the number of instances of the minority class grew up from 1,983 to 9,915.

Regarding sampsize, three different approaches were considered (**Supplementary Figure S2**): using the default value of the randomForest function, equaling the number of cases and controls (50% / 50%); and reducing the number of controls by averaging them, as it was proposed by Chicco et al[2](67% / 33%), according to the next mathematical expression:

$$Sampsize controls number=\frac{\left( 1-Cases\_Control proportion \right) x No cases}{\left( Cases\_Control proportion \right)}$$

Where:

$$Cases\_Control proportion=\frac{\left( \frac{No cases}{No cases+No controles} \right)+0.5}{2}$$

Feature selection and dimensionality reduction were assessed under two different approaches: VIMP and PCA.

Regarding VIMP, this parameter compares a variable's predictive power to its power under randomness. The closer to zero, the lower the predictive capacity, as the difference between the predictive power of the variable and its predictive power under randomness is small. Based on the relative VIMP (obtained by dividing the VIMP score assigned to a specific variable by the VIMP score assigned to the first ranked variable), we selected all variables, or those with a relative VIMP greater that 1%, 5%, 10%, 20% or 50%.

Regarding PCA, the predictors of the dataset are transformed into a set of uncorrelated variables called principal components. Depending on the CP of variance of principal components, we selected those until the desired CP was reached. 0.25, 0.4, 0.5, 0.6, 0.7, 0.8, 0.9 values were tested.

Model calibration was performed in a two-step process. In the first process, we developed prediction models using a particular combination of ntree, mtry, SMOTE and sampsize, which included all available variables. Then, in a second step, the number of predictors was reduced based on the relative VIMP. We used the same ntree and sampsize from the parent model from the first step. Regarding mtry, its value was adjusted to the new number of variables, also testing the new default value ±5 and ±10. The theoretical maximum number of models to be developed based on the combination of tuning parameters was:

$\left[ 4 \left( ntree \right)*5 \left( mtry \right)*3 \left( sampsize \right) * 31 \left( \mathrm{SMOTE} \right)* 5 \left( \mathrm{VIMP} \right)*5 \left( \mathrm{mtry}^{'} \right) \right]+\left[ 4 \left( ntree \right)* 5 \left( mtry \right)* 3 \left( sampsize \right)*31 \left( SMOTE \right) \right]+[4 \left( ntree \right)*5 \left( mtry \right)*3 \left( sampsize \right)*31 \left( SMOTE \right)*7 \left( PCA \right)]$

$$=$$

$4 \left( ntree \right)*5 \left( mtry \right)*3 \left( sampsize \right)* 31 \left( SMOTE \right)*[5\left( \mathrm{VIMP} \right)*5\left( \mathrm{mtry}^{'} \right)+1+7]$

$$=61,380 models$$

To avoid common errors derived from over and under-sampling techniques when performing cross-validation, SMOTE was carried out after splitting the training dataset[5].

**Methods**

Tree based methods are known for being high-variance and low bias. Random Forest generates multiple decision de-correlated trees based on bootstrap data from the original sample and predicts the classification outcome of interest based on the majority votes of the individual decision trees. Therefore, this method allows capturing complex interactions structures in the data, if trees are grown enough, while reducing the variance by bagging individual trees which estimations are further ensemble. Briefly, the algorithm can be described as follows:

1. Starting from the original data, *n* bootstrap samples are drawn. Each sample is randomly divided in two: in-bag data and out-of-bag data (OOB data).

2. From each bootstrap sample, a decision tree is grown using the in-bag data. At each node of the tree, a number of candidate variables (commonly equal to the squared root number of candidate variables) are randomly selected, to reduce the correlation among trees (and variance), and the candidate variable that maximizes difference between the two daughter nodes is used for splitting the node.

3. The tree is grown until any of the daughter nodes have less than a certain number of events (in our case 1).

4. The predicted outcome is determined based on the majority votes of the individual decision trees

We measured the area under the receiver operating characteristic curve (AUC-ROC) by predicting the probability of each particular discharge episode of being followed by an outpatient readmission. The AUC is the probability that in two randomly selected pairs of discharges (one with a following readmission, one without), the classifier will rank higher the discharge followed by readmission[6]. It ranges from 0.5 (non-informative) to 1.0 (perfect discrimination).

Calibration was measured using calibration curves that plot the predicted proportion of discharges with readmission, and the observed ones, for intervals with different predicted risks.

**Supplementary References**

1. Leon, L.; Rodriguez-Rodriguez, L.; Aguilar, M.D.; Jover, J.Á.; Vadillo, C.; Redondo, M.; Abasolo, L. Validation of a Quality of Life Instrument in Spanish Patients With Rheumatic Diseases: The Rosser Classification System. *J. Clin. Rheumatol.* **2018**.

2. Chicco, D. Ten quick tips for machine learning in computational biology. *BioData Min.* **2017**, *10*, 35.

3. *Pattern Recognition and Image Analysis*; Vitrià, J., Sanches, J.M., Hernández, M., Eds.; Lecture Notes in Computer Science; Springer Berlin Heidelberg: Berlin, Heidelberg, 2011; Vol. 6669; ISBN 978-3-642-21256-7.

4. Torgo, L. Data Mining with R, learning with case studies.

5. Blagus, R.; Lusa, L. Joint use of over- and under-sampling techniques and cross-validation for the development and assessment of prediction models. *BMC Bioinformatics* **2015**, *16*, 363.

6. Rahimian, F.; Salimi-Khorshidi, G.; Payberah, A.H.; Tran, J.; Ayala Solares, R.; Raimondi, F.; Nazarzadeh, M.; Canoy, D.; Rahimi, K. Predicting the risk of emergency admission with machine learning: Development and validation using linked electronic health records. *PLoS Med.* **2018**, *15*, e1002695.
