# Supplementary material for: Outpatient Readmission in Rheumatology: A Machine Learning Predictive Model of Patient’s Return to the Clinic
Source: J Clin Med. 2019 Aug 2;8(8):1156. doi: 10.3390/jcm8081156 (PMC6723392; doi:10.3390/jcm8081156)
Supplement: Supplementary file 1 [file jcm-08-01156-s001.zip › Supplementary_File_Tables_v2_R2_v3.docx]

**Supplementary Table S1.** Tuning parameters considered to develop a Random Forest prediction model of readmission in a rheumatology outpatient clinic.

| Parameter | Description | Values |
| --- | --- | --- |
| ntree | Number of trees to grow | 100, 250, 500, 1000 |
| mtry | Number of variables randomly sampled as candidates at each split | $\sqrt{P}-10 ,\sqrt{P}-5,\sqrt{P}, \sqrt{P}+5, \sqrt{P}+10$ |
| SMOTE | k: k-nearest neighbors of each example of the minority class to be used to create new instances  perc.over: Number of instances of the minority class to be created | Original training dataset  k: 1, 2, 3, 5, 7, 9  perc.over: 100, 200, 300, 400 |
| sampsize | Size of the sample to grow | Default (number of discharges), 50/50, 67/33 |
| relative VIMP | Importance of predictors of the main model. Used to build a reduced model | All variables, rVIMP > 1 rVIMP >, 5, rVIMP > 10, rVIMP > 20, rVIMP > 50 |
| mtry’ | Number of variables randomly sampled as candidates at each split | $\sqrt{P^{'}}-10 ,\sqrt{P^{'}}-5,\sqrt{P^{'}}, \sqrt{P^{'}}+5, \sqrt{P^{'}}+10$ |
| PCA | Cumulative proportion of variance of principal components | CP: 0.25, 0.4, 0.5, 0.6, 0.7, 0.8, 0.9 |
|  |  |  |

| *perc.over* | *perc.under* | Number of episodes without outpatient readmission | Number of episodes with outpatient readmission |
| --- | --- | --- | --- |
| 100 | 554.11 | 10,988 | 3,966 |
| 200 | 277.05 | 10,988 | 5,949 |
| 300 | 184.70 | 10,988 | 7,932 |
| 400 | 138.53 | 10,988 | 9,915 |
|  |  |  |  |

**Supplementary Table S2**: Values of over and under sampling used in the Synthetic Minority Over-Sampling Technique (SMOTE) in the training dataset.

**Supplementary Table S3**: Sensibility and specificity for the “discharge status” variable.

|  | Sensitivity | Specificity |
| --- | --- | --- |
| All reviewed visits | 0.805 | 0.709 |
| Reviewed visits with no return | 0.857 | 0.684 |
| Reviewed visits with return, anytime | 0.718 | 0.892 |
| Return in the first 12 months | 0.825 | 0.693 |
| Return in the first 12 months excluding the first month | 0.937 | 0.672 |
| Return in the first 12 months excluding the first 2 months | 0.935 | 0.690 |
| Return in the first 12 months excluding the first 3 months | 0.931 | 0.695 |
| Return in the first 12 months excluding the first 4 months | 0.927 | 0.714 |
| Return in the first 12 months excluding the first 5 months | 0.924 | 0.729 |
| Return in the first 12 months excluding the first 6 months | 0.913 | 0.753 |
| Return in the first 12 months excluding the first 7 months | 0.919 | 0.722 |
| Return in the first 12 months excluding the first 8 months | 0.922 | 0.742 |
| Return in the first 12 months excluding the first 9 months | 0.921 | 0.728 |
| Return in the first 12 months excluding the first 10 months | 0.905 | 0.725 |
| Return in the first 12 months excluding the first 11 months | 0.886 | 0.771 |
|  |  |  |

**Supplementary Table S4**: Demographic and clinical-related characteristics at first visit in the *Hospital Clínico San Carlos* (HCSC) rheumatology outpatient clinic of the patients from the HCSC musculoskeletal cohort used to develop a prediction model of outpatient readmission, based on their readmission or not to the clinic. Differences expressed as Standardized Mean Differences.

| Variables | With outpatient readmission  n = 2,528 | Without outpatient readmission  n = 16,134 | Standardized Mean Difference |
| --- | --- | --- | --- |
| Age in years, median (IQR) | 59.8 (48 - 73.2) | 55.4 (43.9 - 69.4) | 0.205 |
| Distress, n (%) |  |  | 0.193 |
| None | 322 (12.74) | 2,994 (18.56) | - |
| Low | 1,678 (66.38) | 10,615 (65.79) | - |
| Moderate | 510 (20.17) | 2,423 (15.02) | - |
| High | 18 (0.71) | 103 (0.64) | - |
| Disability, n (%) |  |  | 0.300 |
| None | 730 (28.88) | 6,897 (42.75) | - |
| Slight social | 1,229 (48.62) | 6,364 (39.44) | - |
| Severe social and slight physical | 331 (13.09) | 1,715 (10.63) | - |
| Moderate decrease in mobility | 163 (6.45) | 708 (4.39) | - |
| Severe decrease in mobility | 63 (2.49) | 334 (2.07) | - |
| Almost dependent | 12 (0.47) | 110 (0.68) | - |
| In bed | - | 7 (0.04) | - |
| Rosser Index, median (IQR) | 98.6 (97.3 - 99.5) | 98.6 (98.6 - 99.5) |  |
| Occupation, n (%) | - | - | 0.179 |
| Active | 1,278 (50.55) | 9,248 (57.32) | - |
| Housework | 707 (27.97) | 3,671 (22.75) | - |
| Retired | 511 (20.21) | 2,807 (17.40) | - |
| Student | 32 (1.27) | 409 (2.54) | - |
| Diagnosis, n (%) | - | - | - |
| Axial neuropathy | 81 (3.2) | 443 (2.75) | 0.027 |
| Back pain | 286 (11.31) | 2,646 (16.4) | 0.148 |
| Chronic polyarthritis | 210 (8.31) | 179 (1.11) | 0.345 |
| Crystal arthropathy | 92 (3.64) | 450 (2.79) | 0.048 |
| Fibromyalgia | 42 (1.66) | 167 (1.04) | 0.054 |
| Gout | 71 (2.81) | 291 (1.8) | 0.067 |
| Muscle disorders | 68 (2.69) | 698 (4.33) | 0.089 |
| Neck pain | 128 (5.06) | 1,096 (6.79) | 0.073 |
| No diagnoses | 264 (10.44) | 2,276 (14.11) | 0.112 |
| Generalized or Unspecified Osteoarthritis | 173 (6.84) | 1,064 (6.59) | 0.01 |
| Osteoarthritis of first carpometacarpal joints | 51 (2.02) | 386 (2.39) | 0.026 |
| Osteoarthritis of hand | 81 (3.2) | 865 (5.36) | 0.107 |
| Osteoarthritis of hip | 40 (1.58) | 275 (1.7) | 0.01 |
| Osteoarthritis of knee | 239 (9.45) | 1,469 (9.1) | 0.012 |
| Osteoporosis | 73 (2.89) | 346 (2.14) | 0.047 |
| Osteoporosis fracture | 69 (2.73) | 222 (1.38) | 0.096 |
| Other connective tissue inflammatory diseases | 101 (4) | 211 (1.31) | 0.168 |
| Other non-inflammatory diseases | 33 (1.31) | 379 (2.35) | 0.078 |
| Pain in joint | 167 (6.61) | 1,965 (12.18) | 0.192 |
| Peripheral neuropathy | 84 (3.32) | 414 (2.57) | 0.045 |
| Spondyloarthropathies | 56 (2.22) | 77 (0.48) | 0.151 |
| Tendinitis | 93 (3.68) | 727 (4.51) | 0.042 |
| Tendinitis lower extremities | 227 (8.98) | 1,113 (6.9) | 0.077 |
| Tendinitis upper extremities | 446 (17.64) | 2,571 (15.94) | 0.046 |
| Treatment, n (%) | - | - | - |
| Analgesic 1st level | 835 (33.03) | 5,434 (33.68) | 0.014 |
| Analgesic 2nd and 3rd level | 245 (9.69) | 1,165 (7.22) | 0.089 |
| Antidepressant | 38 (1.5) | 162 (1) | 0.045 |
| Antiosteoporotic | 79 (3.12) | 288 (1.79) | 0.087 |
| Benzodiazepine | 158 (6.25) | 1,210 (7.5) | 0.049 |
| Calcium and vitamin D | 196 (7.75) | 727 (4.51) | 0.136 |
| Colchicine | 50 (1.98) | 192 (1.19) | 0.063 |
| Corticosteroid | 288 (11.39) | 630 (3.9) | 0.285 |
| Gabapentin | 47 (1.86) | 267 (1.65) | 0.016 |
| Gastric protector | 405 (16.02) | 2,179 (13.51) | 0.071 |
| Lowering uric acid drugs | 22 (0.87) | 88 (0.55) | 0.039 |
| NSAIDs | 781 (30.89) | 5,141 (31.86) | 0.021 |
| NSAIDs hard | 39 (1.54) | 166 (1.03) | 0.046 |
| Other DMARDs | 105 (4.15) | 29 (0.18) | 0.276 |
| Other drugs | 37 (1.46) | 186 (1.15) | 0.027 |
| SYSADOA | 35 (1.38) | 296 (1.83) | 0.036 |
|  |  |  |  |

NSAIDs: Nonsteroidal anti-inflammatory drugs; SYSADOA: Symptomatic Slow Action Drugs for Osteoarthritis; DMARDs: Disease-Modifying Anti-rheumatic drugs.

**Supplementary Table S5**: Clinical-related characteristics at the first visit of each episode, of the patients included in the *Hospital Clínico San Carlos* musculoskeletal cohort, based on their readmission or not to the clinic. Differences expressed as Standardized Mean Differences.

| Variables | With outpatient readmission  n = 2,528 | Without outpatient readmission  n = 16,134 | Standardized Mean Difference |
| --- | --- | --- | --- |
| Distress, n (%) |  |  | 0.189 |
| None | 324 (12.82) | 2,987 (18.51) |  |
| Low | 1,671 (66.10) | 10,588 (65.63) |  |
| Moderate | 514 (20.33) | 2,462 (15.26) |  |
| High | 19 (0.75) | 97 (0.60) |  |
| Disability, n (%) |  |  | 0.295 |
| None | 735 (29.07) | 6,896 (42.74) |  |
| Slight social | 1,234 (48.81) | 6,348 (39.35) |  |
| Severe social and slight physical | 352 (13.92) | 1,740 (10.78) |  |
| Moderate decrease in mobility | 141 (5.58) | 689 (4.27) |  |
| Severe decrease in mobility | 53 (2.10) | 341 (2.11) |  |
| Almost dependent | 13 (0.51) | 113 (0.7) |  |
| In bed | 0 | 7 (0.04) |  |
| Rosser Index, median (IQR) | 98.6 (97.3-99.5) | 98.6 (98.6-99.5) | 0.041 |
| Occupation, n (%) |  |  | 0.179 |
| Active | 1,270 (50.24) | 9,212 (57.10) |  |
| Housework | 708 (28.01) | 3,728 (23.11) |  |
| Retired | 518 (20.49) | 2,790 (17.29) |  |
| Student | 32 (1.27) | 404 (2.50) |  |
| Diagnoses, n (%) | - | - | - |
| Axial neuropathy | 90 (3.56) | 445 (2.76) | 0.046 |
| Back pain | 286 (11.31) | 2,677 (16.59) | 0.153 |
| Chronic polyarthritis | 234 (9.26) | 181 (1.12) | 0.373 |
| Crystal arthropathy | 93 (3.68) | 452 (2.8) | 0.05 |
| Fibromyalgia | 47 (1.86) | 178 (1.1) | 0.063 |
| Gout | 73 (2.89) | 290 (1.8) | 0.072 |
| Muscle disorders | 64 (2.53) | 701 (4.34) | 0.1 |
| Neck pain | 108 (4.27) | 1,071 (6.64) | 0.104 |
| No diagnoses | 182 (7.2) | 2,154 (13.35) | 0.204 |
| Generalized or Unspecified Osteoarthritis | 172 (6.8) | 1,074 (6.66) | 0.006 |
| Osteoarthritis of first carpometacarpal joints | 53 (2.1) | 383 (2.37) | 0.019 |
| Osteoarthritis of hand | 82 (3.24) | 869 (5.39) | 0.106 |
| Osteoarthritis of hip | 42 (1.66) | 293 (1.82) | 0.012 |
| Osteoarthritis of knee | 279 (11.04) | 1,536 (9.52) | 0.05 |
| Osteoporosis | 74 (2.93) | 359 (2.23) | 0.044 |
| Osteoporosis fracture | 77 (3.05) | 232 (1.44) | 0.109 |
| Other connective tissue inflammatory diseases | 121 (4.79) | 214 (1.33) | 0.202 |
| Other non-inflammatory diseases | 43 (1.7) | 396 (2.45) | 0.053 |
| Pain in joint | 156 (6.17) | 1,953 (12.1) | 0.207 |
| Peripheral neuropathy | 91 (3.6) | 415 (2.57) | 0.059 |
| Spondyloarthropathies | 70 (2.77) | 77 (0.48) | 0.182 |
| Tendinitis | 99 (3.92) | 712 (4.41) | 0.025 |
| Tendinitis lower extremities | 237 (9.38) | 1,175 (7.28) | 0.076 |
| Tendinitis upper extremities | 455 (18) | 2,591 (16.06) | 0.052 |
| Treatment, n (%) | 90 (3.56) | - | - |
| Analgesic 1st level | 921 (36.43) | 5,658 (35.07) | 0.028 |
| Analgesic 2nd and 3rd level | 284 (11.23) | 1,274 (7.9) | 0.114 |
| Antidepressant | 48 (1.9) | 184 (1.14) | 0.062 |
| Antiosteoporotic | 116 (4.59) | 325 (2.01) | 0.144 |
| Benzodiazepine | 166 (6.57) | 1,227 (7.61) | 0.04 |
| Calcium and vitamin D | 268 (10.6) | 787 (4.88) | 0.215 |
| Colchicine | 67 (2.65) | 208 (1.29) | 0.098 |
| Corticosteroid | 368 (14.56) | 640 (3.97) | 0.372 |
| Gabapentin | 60 (2.37) | 290 (1.8) | 0.04 |
| Gastric protector | 437 (17.29) | 2,225 (13.79) | 0.097 |
| Lowering uric acid drugs | 31 (1.23) | 103 (0.64) | 0.061 |
| NSAIDs | 803 (31.76) | 5,239 (32.47) | 0.015 |
| NSAIDs hard | 40 (1.58) | 161 (1) | 0.052 |
| Other DMARDs | 197 (7.79) | 44 (0.27) | 0.389 |
| Other drugs | 51 (2.02) | 207 (1.28) | 0.058 |
| SYSADOA | 37 (1.46) | 320 (1.98) | 0.04 |
|  |  |  |  |

NSAIDs: Nonsteroidal anti-inflammatory drugs; SYSADOA: Symptomatic Slow Action Drugs for Osteoarthritis; DMARDs: Disease-Modifying Anti-rheumatic drugs.

**Supplementary Table S6**: Demographic and clinical-related characteristics at discharge of the patients included in the *Hospital Clínico San Carlos* musculoskeletal cohort, based on their readmission or not to the clinic. All diseases and treatments were included in this table, except for those included in Table 1. Differences expressed as Standardized Mean Differences.

| Variables | With outpatient readmission  n = 2,528 | Without outpatient readmission  n = 16,134 | Standardized Mean Difference |
| --- | --- | --- | --- |
| Diagnoses, n (%) | - | - | - |
| Axial neuropathy | 92 (3.64) | 448 (2.78) | 0.049 |
| Chronic polyarthritis | 235 (9.3) | 157 (0.97) | 0.384 |
| Crystal arthropathy | 89 (3.52) | 406 (2.52) | 0.059 |
| Fibromyalgia | 57 (2.25) | 192 (1.19) | 0.082 |
| Gout | 74 (2.93) | 295 (1.83) | 0.072 |
| Muscle disorders | 62 (2.45) | 713 (4.42) | 0.108 |
| Neck pain | 111 (4.39) | 1,073 (6.65) | 0.099 |
| Generalized or Unspecified Osteoarthritis | 185 (7.32) | 1,158 (7.18) | 0.005 |
| Osteoarthritis of first carpometacarpal joints | 55 (2.18) | 409 (2.54) | 0.024 |
| Osteoarthritis of hand | 83 (3.28) | 888 (5.5) | 0.109 |
| Osteoarthritis of hip | 51 (2.02) | 321 (1.99) | 0.002 |
| Osteoporosis | 85 (3.36) | 374 (2.32) | 0.063 |
| Osteoporosis fracture | 95 (3.76) | 274 (1.7) | 0.127 |
| Other connective tissue inflammatory diseases | 129 (5.1) | 210 (1.3) | 0.217 |
| Other non-inflammatory diseases | 47 (1.86) | 420 (2.6) | 0.05 |
| Peripheral neuropathy | 86 (3.4) | 424 (2.63) | 0.045 |
| Spondyloarthropathies | 76 (3.01) | 77 (0.48) | 0.194 |
| Tendinitis | 104 (4.11) | 730 (4.52) | 0.02 |
| Treatment, n (%) | - | - | - |
| Antidepressant | 56 (2.22) | 199 (1.23) | 0.075 |
| Antiosteoporotic | 159 (6.29) | 389 (2.41) | 0.191 |
| Colchicine | 74 (2.93) | 207 (1.28) | 0.115 |
| Corticosteroid | 353 (13.96) | 482 (2.99) | 0.402 |
| Gabapentin | 71 (2.81) | 314 (1.95) | 0.057 |
| Lowering uric acid drugs | 42 (1.66) | 149 (0.92) | 0.065 |
| NSAIDs hard | 39 (1.54) | 149 (0.92) | 0.056 |
| Other DMARDs | 246 (9.73) | 51 (0.32) | 0.441 |
| Other drugs | 73 (2.89) | 229 (1.42) | 0.101 |
| SYSADOA | 45 (1.78) | 365 (2.26) | 0.034 |
| Other |  |  |  |
| Follow-up days until discharge since last discharge | 0 (0 - 75) | 0 (0 - 0) | 0.175 |
| Number of diagnoses given in each discharge | 1 (1 – 1) | 1 (1 – 1) | 0.060 |
| Number of treatments given in each discharge | 2 (1 - 3) | 1 (0 - 2) | 0.393 |
| Number of discharges given until current discharge | 1 (1 – 2) | 1 (1-1) | 0.505 |
| Number of visits since the last time a discharge was given, until new discharge. If there is no previous discharge, it is the number of visits until discharge, from first visit in clinic (relative visit number) | 1 (1-2) | 1 (1-2) | 0.334 |
| Number of visits from first visit in the outpatient clinic until discharge. | 2 (1-4) | 1 (1-2) | 0.530 |
|  |  |  |  |

NSAIDs: Nonsteroidal anti-inflammatory drugs; SYSADOA: Symptomatic Slow Action Drugs for Osteoarthritis; DMARDs: Disease-Modifying Anti-rheumatic drugs

**Supplementary Table S7**: Mean and median values of the Rosser Classification Index, and disability and pain/distress subscales, of the visits carried out in the last 90 or 182 days before discharge, of the patients included in the *Hospital Clínico San Carlos* musculoskeletal cohort, based on their readmission or not to the clinic. Differences expressed as Standardized Mean Differences.

| Variables | With outpatient readmission  n = 2,528 | Without outpatient readmission  n = 16,134 | Standardized Mean Difference |
| --- | --- | --- | --- |
| Distress, median (IQR) | - | - | - |
| 90c | 1 (1-1) | 1 (1-1) | 0.161 |
| 90w | 1 (1-1) | 1 (1-1) | 0.154 |
| 182c | 1 (1-1) | 1 (1-1) | 0.154 |
| 182w | 1 (1-1) | 1 (1-1) | 0.142 |
| Disability, median (IQR) | - | - | - |
| 90c | 2 (1-2) | 2 (1-2) | 0.154 |
| 90w | 2 (1-2) | 2 (1-2) | 0.151 |
| 182c | 2 (1-2) | 2 (1-2) | 0.153 |
| 182w | 2 (1-2) | 2 (1-2) | 0.152 |
| Rosser, median (IQR) | - | - | - |
| 90c | 0.986  (0.976 – 0.995) | 0.986  (0.986 – 0.995) | 0.026 |
| 90w | 0.986  (0.973-0.995) | 0.986  (0.986-0.995) | 0.028 |
| 182c | 0.986  (0.976 – 0.995) | 0.986  (0.986-0.995) | 0.026 |
| 182w | 0.986  (0.973-0.995) | 0.986  (0.986-0.995) | 0.028 |
| Distress, mean | - | - | - |
| 90c | 1 (1-1) | 1 (1-1) | 0.157 |
| 90w | 1 (1-1) | 1 (1-1) | 0.152 |
| 182c | 1 (1-1) | 1 (1-1) | 0.155 |
| 182w | 1 (1-1) | 1 (1-1) | 0.142 |
| Disability, mean | - | - | - |
| 90c | 2 (1-2) | 2 (1-2) | 0.150 |
| 90w | 2 (1-2) | 2 (1-2) | 0.149 |
| 182c | 2 (1-2) | 2 (1-2) | 0.155 |
| 182w | 2 (1-2) | 2 (1-2) | 0.151 |
| Rosser, mean | - | - | - |
| 90c | 0.986  (0.977 – 0.995) | 0.986  (0.986 – 0.995) | 0.025 |
| 90w | 0.986  (0.973-0.995) | 0.986  (0.986-0.995) | 0.027 |
| 182c | 0.986  (0.976 – 0.995) | 0.986  (0.986 – 0.995) | 0.028 |
| 182w | 0.986  (0.973-0.995) | 0.986  (0.986-0.995) | 0.030 |
|  |  |  |  |

90c: health status information in the last 90 days, including current discharge visit. 90w: health status information in the last 90 days, excluding current discharge visit. 182c: health status information in the last 182 days, including current discharge visit. 182w: health status information in the last 182 days, excluding current discharge visit.

**Supplementary Table S8**: Diagnosis given, and treatment prescribed during the visits taking place in the 90 days before discharge of the patients included in the *Hospital Clínico San Carlos* musculoskeletal cohort, based on their readmission or not to the clinic. Differences expressed as Standardized Mean Differences.

| Variables | With outpatient readmission  n = 2,528 | Without outpatient readmission  n = 16,134 | Standardized Mean Difference |
| --- | --- | --- | --- |
| Diagnoses, n (%) | - | - | - |
| Axial neuropathy | 96 (3.8) | 477 (2.96) | 0.047 |
| Back pain | 343 (13.57) | 2,829 (17.53) | 0.11 |
| Chronic polyarthritis | 249 (9.85) | 175 (1.08) | 0.393 |
| Crystal arthropathy | 103 (4.07) | 474 (2.94) | 0.062 |
| Fibromyalgia | 58 (2.29) | 197 (1.22) | 0.082 |
| Gout | 75 (2.97) | 307 (1.9) | 0.069 |
| Muscle disorders | 71 (2.81) | 738 (4.57) | 0.094 |
| Neck pain | 116 (4.59) | 1,104 (6.84) | 0.097 |
| No diagnoses | 164 (6.49) | 2,129 (13.2) | 0.227 |
| Generalized or Unspecified Osteoarthritis | 200 (7.91) | 1,183 (7.33) | 0.022 |
| Osteoarthritis of first carpometacarpal joints | 61 (2.41) | 421 (2.61) | 0.013 |
| Osteoarthritis of hand | 87 (3.44) | 910 (5.64) | 0.106 |
| Osteoarthritis of hip | 56 (2.22) | 333 (2.06) | 0.01 |
| Osteoarthritis of knee | 316 (12.5) | 1,706 (10.57) | 0.06 |
| Osteoporosis | 89 (3.52) | 385 (2.39) | 0.067 |
| Osteoporosis fracture | 99 (3.92) | 278 (1.72) | 0.133 |
| Other connective tissue inflammatory diseases | 130 (5.14) | 222 (1.38) | 0.213 |
| Other non-inflammatory diseases | 55 (2.18) | 455 (2.82) | 0.041 |
| Pain in joint | 161 (6.37) | 2,035 (12.61) | 0.214 |
| Peripheral neuropathy | 92 (3.64) | 457 (2.83) | 0.046 |
| Spondyloarthropathies | 79 (3.12) | 90 (0.56) | 0.192 |
| Tendinitis | 113 (4.47) | 778 (4.82) | 0.017 |
| Tendinitis lower extremities | 264 (10.44) | 1,253 (7.77) | 0.093 |
| Tendinitis upper extremities | 494 (19.54) | 2,680 (16.61) | 0.076 |
| Treatment, n (%) | - | - | - |
| Analgesic 1st level | 1,048 (41.46) | 6,101 (37.81) | 0.074 |
| Analgesic 2nd and 3rd level | 345 (13.65) | 1,450 (8.99) | 0.147 |
| Antidepressant | 58 (2.29) | 205 (1.27) | 0.077 |
| Antiosteoporotic | 160 (6.33) | 399 (2.47) | 0.189 |
| Benzodiazepine | 178 (7.04) | 1,268 (7.86) | 0.031 |
| Calcium and vitamin D | 355 (14.04) | 1,027 (6.37) | 0.256 |
| Colchicine | 79 (3.12) | 227 (1.41) | 0.116 |
| Corticosteroid | 396 (15.66) | 635 (3.94) | 0.402 |
| Gabapentin | 77 (3.05) | 342 (2.12) | 0.058 |
| Gastric protector | 492 (19.46) | 2,260 (14.01) | 0.147 |
| Lowering uric acid drugs | 42 (1.66) | 149 (0.92) | 0.065 |
| NSAIDs | 885 (35.01) | 5,462 (33.85) | 0.024 |
| NSAIDs hard | 42 (1.66) | 163 (1.01) | 0.057 |
| Other DMARDs | 249 (9.85) | 61 (0.38) | 0.44 |
| Other drugs | 85 (3.36) | 263 (1.63) | 0.111 |
| SYSADOA | 46 (1.82) | 367 (2.27) | 0.032 |
|  |  |  |  |

NSAIDs: Nonsteroidal anti-inflammatory drugs; SYSADOA: Symptomatic Slow Action Drugs for Osteoarthritis; DMARDs: Disease-Modifying Antirheumatic drugs

**Supplementary Table S9**: Diagnosis given, and treatment prescribed during the visits taking place in the 182 days before discharge of the patients included in the *Hospital Clínico San Carlos* musculoskeletal cohort, based on their readmission or not to the clinic. Differences expressed as Standardized Mean Differences.

| Variables | With outpatient readmission  n = 2,528 | Without outpatient readmission  n = 16,134 | Standardized Mean Difference |
| --- | --- | --- | --- |
| Diagnoses, n (%) | - | - | - |
| Axial neuropathy | 97 (3.84) | 491 (3.04) | 0.044 |
| Back pain | 353 (13.96) | 2,855 (17.7) | 0.102 |
| Chronic polyarthritis | 256 (10.13) | 184 (1.14) | 0.397 |
| Crystal arthropathy | 106 (4.19) | 488 (3.02) | 0.063 |
| Fibromyalgia | 59 (2.33) | 199 (1.23) | 0.083 |
| Gout | 75 (2.97) | 308 (1.91) | 0.069 |
| Muscle disorders | 80 (3.16) | 750 (4.65) | 0.077 |
| Neck pain | 121 (4.79) | 1,120 (6.94) | 0.092 |
| No diagnoses | 188 (7.44) | 2,198 (13.62) | 0.203 |
| Generalized or Unspecified Osteoarthritis | 206 (8.15) | 1,192 (7.39) | 0.028 |
| Osteoarthritis of first carpometacarpal joints | 62 (2.45) | 425 (2.63) | 0.012 |
| Osteoarthritis of hand | 91 (3.6) | 915 (5.67) | 0.099 |
| Osteoarthritis of hip | 59 (2.33) | 338 (2.09) | 0.016 |
| Osteoarthritis of knee | 319 (12.62) | 1,716 (10.64) | 0.062 |
| Osteoporosis | 89 (3.52) | 388 (2.4) | 0.066 |
| Osteoporosis fracture | 100 (3.96) | 281 (1.74) | 0.133 |
| Other connective tissue inflammatory diseases | 132 (5.22) | 230 (1.43) | 0.213 |
| Other non-inflammatory diseases | 56 (2.22) | 462 (2.86) | 0.041 |
| Pain in joint | 173 (6.84) | 2,085 (12.92) | 0.205 |
| Peripheral neuropathy | 96 (3.8) | 474 (2.94) | 0.048 |
| Spondyloarthropathies | 82 (3.24) | 96 (0.6) | 0.194 |
| Tendinitis | 117 (4.63) | 791 (4.9) | 0.013 |
| Tendinitis lower extremities | 273 (10.8) | 1,290 (8) | 0.096 |
| Tendinitis upper extremities | 499 (19.74) | 2,710 (16.8) | 0.076 |
| Treatment, n (%) |  |  |  |
| Analgesic 1st level | 1,058 (41.85) | 6,151 (38.12) | 0.076 |
| Analgesic 2nd and 3rd level | 360 (14.24) | 1,478 (9.16) | 0.159 |
| Antidepressant | 60 (2.37) | 215 (1.33) | 0.077 |
| Antiosteoporotic | 166 (6.57) | 402 (2.49) | 0.197 |
| Benzodiazepine | 191 (7.56) | 1,300 (8.06) | 0.019 |
| Calcium and vitamin D | 364 (14.4) | 1,042 (6.46) | 0.262 |
| Colchicine | 84 (3.32) | 245 (1.52) | 0.118 |
| Corticosteroid | 421 (16.65) | 692 (4.29) | 0.412 |
| Gabapentin | 81 (3.2) | 358 (2.22) | 0.061 |
| Gastric protector | 508 (20.09) | 2,310 (14.32) | 0.154 |
| Lowering uric acid drugs | 43 (1.7) | 150 (0.93) | 0.068 |
| NSAIDs | 911 (36.04) | 5,562 (34.47) | 0.033 |
| NSAIDs hard | 46 (1.82) | 170 (1.05) | 0.064 |
| Other DMARDs | 254 (10.05) | 69 (0.43) | 0.442 |
| Other drugs | 92 (3.64) | 275 (1.7) | 0.12 |
| SYSADOA | 47 (1.86) | 371 (2.3) | 0.031 |
|  |  |  |  |

NSAIDs: Nonsteroidal anti-inflammatory drugs; SYSADOA: Symptomatic Slow Action Drugs for Osteoarthritis; DMARDs: Disease-Modifying Anti-rheumatic drugs

**Supplementary Table S10**: Comorbidities and concomitant medication present at discharge, of the patients included in the *Hospital Clínico San Carlos* musculoskeletal cohort, based on their readmission or not to the clinic. All comorbidities were included in this table, except for those included in Table 1. Differences expressed as Standardized Mean Differences.

| Variables | With outpatient readmission  n = 2,528 | Without outpatient readmission  n = 16,134 | Standardized Mean Difference |
| --- | --- | --- | --- |
| Comorbidities and concomitant treatments, n (%) |  |  |  |
| Alcoholic habit | 37 (1.46) | 272 (1.69) | 0.018 |
| Allergy | 54 (2.14) | 289 (1.79) | 0.025 |
| Allergy medication | 26 (1.03) | 154 (0.95) | 0.007 |
| Alpha-blocker | 21 (0.83) | 124 (0.77) | 0.007 |
| Ambiental allergy | 47 (1.86) | 273 (1.69) | 0.013 |
| Anemia | 35 (1.38) | 189 (1.17) | 0.019 |
| Angiotensin II receptor blocker | 212 (8.39) | 1,122 (6.95) | 0.054 |
| Angiotensin-converting-enzyme inhibitor | 238 (9.41) | 1,242 (7.7) | 0.061 |
| Antiaggregant | 216 (8.54) | 939 (5.82) | 0.106 |
| Anticoagulant | 87 (3.44) | 501 (3.11) | 0.019 |
| Antidepressant | 198 (7.83) | 1,059 (6.56) | 0.049 |
| Antiepileptics | 31 (1.23) | 139 (0.86) | 0.036 |
| Anxiety | 48 (1.9) | 309 (1.92) | 0.001 |
| Anxiety medication | 228 (9.02) | 1,120 (6.94) | 0.077 |
| Appendicitis | 61 (2.41) | 440 (2.73) | 0.02 |
| Articular prosthesis | 94 (3.72) | 488 (3.02) | 0.038 |
| Articular surgery no prosthesis | 14 (0.55) | 89 (0.55) | 0 |
| Asthma | 69 (2.73) | 361 (2.24) | 0.032 |
| Atrial fibrillation | 51 (2.02) | 340 (2.11) | 0.006 |
| Axial neuropathy | 28 (1.11) | 98 (0.61) | 0.054 |
| Back pain | 78 (3.09) | 392 (2.43) | 0.04 |
| Benign breast tumor | 26 (1.03) | 148 (0.92) | 0.011 |
| Benign prostatic hyperplasia medication | 49 (1.94) | 243 (1.51) | 0.033 |
| Benign prostatic hypertrophy | 50 (1.98) | 363 (2.25) | 0.019 |
| Benzodiazepine | 224 (8.86) | 1,091 (6.76) | 0.078 |
| Beta-blocker | 138 (5.46) | 707 (4.38) | 0.05 |
| Bisphosphonates | 80 (3.16) | 425 (2.63) | 0.032 |
| Breast cancer | 40 (1.58) | 235 (1.46) | 0.01 |
| Bronchodilator | 59 (2.33) | 305 (1.89) | 0.031 |
| Calcium | 177 (7) | 831 (5.15) | 0.078 |
| Calcium antagonist | 139 (5.5) | 762 (4.72) | 0.035 |
| Cancer | 68 (2.69) | 436 (2.7) | 0.001 |
| Cancer medication | 45 (1.78) | 218 (1.35) | 0.035 |
| Cataract | 65 (2.57) | 305 (1.89) | 0.046 |
| Central nervous system diseases | 35 (1.38) | 266 (1.65) | 0.022 |
| Cerebrovascular disease | 37 (1.46) | 222 (1.38) | 0.007 |
| Cervicalgia | 27 (1.07) | 151 (0.94) | 0.013 |
| Cholecystectomy | 85 (3.36) | 414 (2.57) | 0.047 |
| Chronic obstructive pulmonary disease | 41 (1.62) | 285 (1.77) | 0.011 |
| Cognitive impairment | 14 (0.55) | 100 (0.62) | 0.009 |
| Colon cancer | 20 (0.79) | 91 (0.56) | 0.028 |
| Colon diverticulitis | 22 (0.87) | 105 (0.65) | 0.025 |
| Colon hemorrhoid | 14 (0.55) | 96 (0.6) | 0.005 |
| Colon surgery | 20 (0.79) | 143 (0.89) | 0.01 |
| Column surgery | 62 (2.45) | 271 (1.68) | 0.054 |
| Connective tissue benign tumor | 35 (1.38) | 197 (1.22) | 0.014 |
| Constipation | 27 (1.07) | 152 (0.94) | 0.013 |
| Contraception | 9 (0.36) | 123 (0.76) | 0.055 |
| Cystocele | 22 (0.87) | 106 (0.66) | 0.024 |
| Digoxin | 8 (0.32) | 97 (0.6) | 0.042 |
| Disease modifying antirheumatic drugs | 15 (0.59) | 101 (0.63) | 0.004 |
| Diuretic | 289 (11.43) | 1,449 (8.98) | 0.081 |
| Fall | 16 (0.63) | 99 (0.61) | 0.002 |
| Family history | 23 (0.91) | 98 (0.61) | 0.035 |
| Feet diseases | 59 (2.33) | 323 (2) | 0.023 |
| Gabapentin | 51 (2.02) | 240 (1.49) | 0.04 |
| Gastritis | 24 (0.95) | 173 (1.07) | 0.012 |
| Glaucoma | 39 (1.54) | 162 (1) | 0.048 |
| Gout | 11 (0.44) | 116 (0.72) | 0.037 |
| Hand diseases | 37 (1.46) | 191 (1.18) | 0.024 |
| Helicobacter pylori infection | 20 (0.79) | 99 (0.61) | 0.021 |
| Hematopoietic cancer | 20 (0.79) | 97 (0.6) | 0.023 |
| Hiatal hernia | 71 (2.81) | 415 (2.57) | 0.015 |
| Hip prosthesis | 18 (0.71) | 122 (0.76) | 0.005 |
| Hyperglycemia | 31 (1.23) | 153 (0.95) | 0.027 |
| Hyperthyroidism | 14 (0.55) | 90 (0.56) | 0.001 |
| Hyperuricemia | 29 (1.15) | 212 (1.31) | 0.015 |
| Hysterectomy surgery | 69 (2.73) | 461 (2.86) | 0.008 |
| Inhaled corticosteroid | 53 (2.1) | 271 (1.68) | 0.031 |
| Insomnia | 23 (0.91) | 80 (0.5) | 0.05 |
| Insulin | 58 (2.29) | 309 (1.92) | 0.026 |
| Internal derangement of knee | 43 (1.7) | 234 (1.45) | 0.02 |
| Iron | 28 (1.11) | 140 (0.87) | 0.024 |
| Irritable colon syndrome | 18 (0.71) | 113 (0.7) | 0.001 |
| Ischemic heart disease | 68 (2.69) | 451 (2.8) | 0.006 |
| Kidney failure | 40 (1.58) | 195 (1.21) | 0.032 |
| Knee arthrosis | 15 (0.59) | 126 (0.78) | 0.023 |
| Knee surgery non prosthesis | 53 (2.1) | 279 (1.73) | 0.027 |
| Knee surgery prosthesis | 34 (1.34) | 184 (1.14) | 0.018 |
| Lowering uric acid drugs | 39 (1.54) | 256 (1.59) | 0.004 |
| Macrocytosis | 14 (0.55) | 90 (0.56) | 0.001 |
| Metamizole | 37 (1.46) | 115 (0.71) | 0.072 |
| Migraine | 29 (1.15) | 239 (1.48) | 0.029 |
| Non osteoporotic fracture | 44 (1.74) | 262 (1.62) | 0.009 |
| NSAIDs | 56 (2.22) | 237 (1.47) | 0.056 |
| Ocular hypertension medication | 37 (1.46) | 162 (1) | 0.042 |
| Oral anticoagulant | 85 (3.36) | 496 (3.07) | 0.016 |
| Oral antidiabetics | 198 (7.83) | 976 (6.05) | 0.07 |
| Osteoporosis | 94 (3.72) | 499 (3.09) | 0.034 |
| Osteoporotic fracture | 48 (1.9) | 258 (1.6) | 0.023 |
| Other antihypertensives | 78 (3.09) | 479 (2.97) | 0.007 |
| Other arrhythmias | 89 (3.52) | 555 (3.44) | 0.004 |
| Other articular surgeries | 93 (3.68) | 462 (2.86) | 0.046 |
| Other benign tumors | 40 (1.58) | 169 (1.05) | 0.047 |
| Other biliary diseases | 106 (4.19) | 485 (3.01) | 0.064 |
| Other colon diseases | 42 (1.66) | 196 (1.21) | 0.038 |
| Other dermatological diseases | 39 (1.54) | 235 (1.46) | 0.007 |
| Other digestive diseases | 70 (2.77) | 420 (2.6) | 0.01 |
| Other ear diseases | 35 (1.38) | 193 (1.2) | 0.017 |
| Other endocrine diseases | 53 (2.1) | 228 (1.41) | 0.052 |
| Other esophagus diseases | 58 (2.29) | 362 (2.24) | 0.003 |
| Other eyes diseases | 39 (1.54) | 217 (1.34) | 0.017 |
| Other heart diseases | 25 (0.99) | 104 (0.64) | 0.038 |
| Other hematologic diseases | 48 (1.9) | 257 (1.59) | 0.023 |
| Other hip diseases | 16 (0.63) | 98 (0.61) | 0.003 |
| Other infection | 62 (2.45) | 296 (1.83) | 0.043 |
| Other knee disease | 21 (0.83) | 88 (0.55) | 0.035 |
| Other liver disease | 35 (1.38) | 195 (1.21) | 0.016 |
| Other musculoskeletal infection | 53 (2.1) | 311 (1.93) | 0.012 |
| Other neurological diseases | 18 (0.71) | 109 (0.68) | 0.004 |
| Other otorhinolaryngology diseases | 41 (1.62) | 277 (1.72) | 0.007 |
| Other ovary diseases | 26 (1.03) | 152 (0.94) | 0.009 |
| Other psychiatric conditions | 25 (0.99) | 138 (0.86) | 0.014 |
| Other respiratory diseases | 18 (0.71) | 99 (0.61) | 0.012 |
| Other spine diseases | 33 (1.31) | 185 (1.15) | 0.014 |
| Other urological diseases | 12 (0.47) | 109 (0.68) | 0.027 |
| Other uterus diseases | 72 (2.85) | 389 (2.41) | 0.027 |
| Other valve diseases | 36 (1.42) | 177 (1.1) | 0.029 |
| Other virus infection | 60 (2.37) | 358 (2.22) | 0.01 |
| Paracetamol | 90 (3.56) | 396 (2.45) | 0.065 |
| Parkinson's disease | 10 (0.4) | 92 (0.57) | 0.025 |
| Peptic ulcer | 43 (1.7) | 227 (1.41) | 0.024 |
| Peripheral artery disease | 16 (0.63) | 118 (0.73) | 0.012 |
| Peripheral nervous system diseases | 80 (3.16) | 358 (2.22) | 0.058 |
| Pregnancy | 35 (1.38) | 312 (1.93) | 0.043 |
| Prostate cancer | 20 (0.79) | 97 (0.6) | 0.023 |
| Proton-pump inhibitor medication | 255 (10.09) | 1,343 (8.32) | 0.061 |
| Psoriasis | 41 (1.62) | 185 (1.15) | 0.041 |
| Pulmonary embolism or deep vein thrombosis | 29 (1.15) | 105 (0.65) | 0.053 |
| Salpingectomy and oophorectomy | 38 (1.5) | 245 (1.52) | 0.001 |
| Second and third analgesia level | 63 (2.49) | 305 (1.89) | 0.041 |
| Shoulder diseases | 28 (1.11) | 148 (0.92) | 0.019 |
| Sleep apnea | 20 (0.79) | 86 (0.53) | 0.032 |
| Smoking habit | 64 (2.53) | 573 (3.55) | 0.059 |
| Statins | 548 (21.68) | 2,807 (17.4) | 0.108 |
| Stomach bowel disease | 16 (0.63) | 91 (0.56) | 0.009 |
| Symptomatic slow action drugs for osteoarthritis | 40 (1.58) | 175 (1.08) | 0.043 |
| Thyroid hormone | 190 (7.52) | 999 (6.19) | 0.052 |
| Thyroid nodule | 11 (0.44) | 98 (0.61) | 0.024 |
| Thyroid others | 22 (0.87) | 137 (0.85) | 0.002 |
| Thyroidectomy | 28 (1.11) | 187 (1.16) | 0.005 |
| Tonsillectomy | 33 (1.31) | 211 (1.31) | 0 |
| Tuberculosis infection | 14 (0.55) | 94 (0.58) | 0.004 |
| Unspecified arthrosis | 23 (0.91) | 130 (0.81) | 0.011 |
| Urological obstruction | 22 (0.87) | 215 (1.33) | 0.044 |
| Urological surgery | 32 (1.27) | 165 (1.02) | 0.023 |
| Venous insufficiency peripheral | 59 (2.33) | 374 (2.32) | 0.001 |
| Viral hepatitis | 29 (1.15) | 215 (1.33) | 0.017 |
| Vitamins | 22 (0.87) | 125 (0.77) | 0.011 |
|  |  |  |  |
|  |  |  |  |

NSAIDs: Nonsteroidal anti-inflammatory drugs; ACE: Angiotensin-converting enzyme; ARBs: Angiotensin II receptor blocker, DMARDs: Disease-Modifying Antirheumatic drugs

**Supplementary Table S11**: Demographic, clinical, and comorbidities-related characteristics at first visit in the clinic, at the first visit of each episode, at discharge, and at any visit taking place in the previous 90 or 182 days before discharge, of the patients included in the *Hospital Clínico San Carlos* musculoskeletal cohort used to develop a prediction model of outpatient readmission, based on their inclusion in the training, validation or test datasets. Differences expressed as Standardized Mean Differences.

| Predictor | TR | VL | TS | TR-VL | TR-TS | VL-TS |
| --- | --- | --- | --- | --- | --- | --- |
| Diagnosis at first visit in clinic |  |  |  |  |  |  |
| Axial neuropathy | 365 (2.81) | 71 (2.68) | 88 (2.89) | 0.008 | 0.005 | 0.012 |
| Back pain | 2,030 (15.65) | 433 (16.37) | 469 (15.4) | 0.02 | 0.007 | 0.027 |
| Chronic polyarthritis | 294 (2.27) | 41 (1.55) | 54 (1.77) | 0.052 | 0.035 | 0.017 |
| Crystal arthropathy | 361 (2.78) | 84 (3.18) | 97 (3.18) | 0.023 | 0.024 | 0 |
| Fibromyalgia | 150 (1.16) | 30 (1.13) | 29 (0.95) | 0.002 | 0.02 | 0.018 |
| Gout | 265 (2.04) | 51 (1.93) | 46 (1.51) | 0.008 | 0.04 | 0.032 |
| Muscle disorders | 531 (4.09) | 111 (4.2) | 124 (4.07) | 0.005 | 0.001 | 0.006 |
| Neck pain | 916 (7.06) | 142 (5.37) | 166 (5.45) | 0.07 | 0.067 | 0.004 |
| No diagnoses | 1,807 (13.93) | 345 (13.04) | 388 (12.74) | 0.026 | 0.035 | 0.009 |
| Generalized or Unspecified Osteoarthritis | 900 (6.94) | 143 (5.41) | 194 (6.37) | 0.064 | 0.023 | 0.041 |
| Osteoarthritis of first carpometacarpal joints | 312 (2.41) | 57 (2.16) | 68 (2.23) | 0.017 | 0.011 | 0.005 |
| Osteoarthritis of hand | 682 (5.26) | 125 (4.73) | 139 (4.56) | 0.024 | 0.032 | 0.008 |
| Osteoarthritis of hip | 212 (1.63) | 46 (1.74) | 57 (1.87) | 0.008 | 0.018 | 0.01 |
| Osteoarthritis of knee | 1,184 (9.13) | 228 (8.62) | 296 (9.72) | 0.018 | 0.02 | 0.038 |
| Osteoporosis | 319 (2.46) | 50 (1.89) | 50 (1.64) | 0.039 | 0.058 | 0.019 |
| Osteoporosis fracture | 216 (1.67) | 35 (1.32) | 40 (1.31) | 0.028 | 0.029 | 0.001 |
| Other connective tissue inflammatory diseases | 227 (1.75) | 43 (1.63) | 42 (1.38) | 0.01 | 0.03 | 0.02 |
| Other non-inflammatory diseases | 242 (1.87) | 72 (2.72) | 98 (3.22) | 0.057 | 0.086 | 0.029 |
| Pain in joint | 1,460 (11.26) | 318 (12.02) | 354 (11.62) | 0.024 | 0.011 | 0.012 |
| Peripheral neuropathy | 335 (2.58) | 65 (2.46) | 98 (3.22) | 0.008 | 0.038 | 0.046 |
| Spondyloarthropathies | 112 (0.86) | 9 (0.34) | 12 (0.39) | 0.068 | 0.059 | 0.009 |
| Tendinitis | 535 (4.12) | 116 (4.39) | 169 (5.55) | 0.013 | 0.066 | 0.054 |
| Tendinitis lower extremities | 866 (6.68) | 226 (8.54) | 248 (8.14) | 0.07 | 0.056 | 0.015 |
| Tendinitis upper extremities | 2,003 (15.44) | 470 (17.77) | 544 (17.86) | 0.063 | 0.065 | 0.002 |
| Treatment at first visit in clinic |  |  |  |  |  |  |
| Analgesic 1st level | 4,388 (33.83) | 878 (33.19) | 1,003 (32.93) | 0.013 | 0.019 | 0.006 |
| Analgesic 2nd and 3rd level | 1,028 (7.93) | 172 (6.5) | 210 (6.89) | 0.055 | 0.039 | 0.016 |
| Antidepressant | 133 (1.03) | 29 (1.1) | 38 (1.25) | 0.007 | 0.021 | 0.014 |
| Antiosteoporotic | 302 (2.33) | 35 (1.32) | 30 (0.98) | 0.075 | 0.105 | 0.032 |
| Benzodiazepine | 1,017 (7.84) | 168 (6.35) | 183 (6.01) | 0.058 | 0.072 | 0.014 |
| Calcium and vitamin D | 675 (5.2) | 128 (4.84) | 120 (3.94) | 0.017 | 0.061 | 0.044 |
| Colchicine | 167 (1.29) | 37 (1.4) | 38 (1.25) | 0.01 | 0.004 | 0.013 |
| Corticosteroid | 644 (4.96) | 122 (4.61) | 152 (4.99) | 0.017 | 0.001 | 0.018 |
| Gabapentin | 205 (1.58) | 52 (1.97) | 57 (1.87) | 0.029 | 0.022 | 0.007 |
| Gastric protector | 1,958 (15.1) | 330 (12.48) | 296 (9.72) | 0.076 | 0.164 | 0.088 |
| Lowering uric acid drugs | 77 (0.59) | 23 (0.87) | 10 (0.33) | 0.032 | 0.039 | 0.07 |
| NSAIDs | 4187 (32.28) | 827 (31.27) | 908 (29.81) | 0.022 | 0.053 | 0.032 |
| NSAIDs hard | 166 (1.28) | 22 (0.83) | 17 (0.56) | 0.044 | 0.076 | 0.033 |
| Other DMARDs | 116 (0.89) | 8 (0.3) | 10 (0.33) | 0.077 | 0.073 | 0.005 |
| Other drugs | 162 (1.25) | 18 (0.68) | 43 (1.41) | 0.058 | 0.014 | 0.072 |
| SYSADOA | 243 (1.87) | 45 (1.7) | 43 (1.41) | 0.013 | 0.036 | 0.023 |
| Diagnosis at discharge |  |  |  |  |  |  |
| Axial neuropathy | 375 (2.89) | 77 (2.91) | 88 (2.89) | 0.001 | 0 | 0.001 |
| Back pain | 2,154 (16.61) | 453 (17.13) | 477 (15.66) | 0.014 | 0.026 | 0.04 |
| Chronic polyarthritis | 325 (2.51) | 29 (1.1) | 38 (1.25) | 0.106 | 0.093 | 0.014 |
| Crystal arthropathy | 333 (2.57) | 78 (2.95) | 84 (2.76) | 0.023 | 0.012 | 0.011 |
| Fibromyalgia | 178 (1.37) | 36 (1.36) | 35 (1.15) | 0.001 | 0.02 | 0.019 |
| Gout | 271 (2.09) | 50 (1.89) | 48 (1.58) | 0.014 | 0.038 | 0.024 |
| Muscle disorders | 551 (4.25) | 114 (4.31) | 110 (3.61) | 0.003 | 0.033 | 0.036 |
| Neck pain | 888 (6.85) | 147 (5.56) | 149 (4.89) | 0.053 | 0.083 | 0.03 |
| No diagnoses | 1,419 (10.94) | 288 (10.89) | 321 (10.54) | 0.002 | 0.013 | 0.011 |
| Generalized or Unspecified Osteoarthritis | 973 (7.5) | 155 (5.86) | 215 (7.06) | 0.066 | 0.017 | 0.049 |
| Osteoarthritis of first carpometacarpal joints | 327 (2.52) | 57 (2.16) | 80 (2.63) | 0.024 | 0.007 | 0.031 |
| Osteoarthritis of hand | 704 (5.43) | 127 (4.8) | 140 (4.6) | 0.028 | 0.038 | 0.01 |
| Osteoarthritis of hip | 249 (1.92) | 55 (2.08) | 68 (2.23) | 0.011 | 0.022 | 0.011 |
| Osteoarthritis of knee | 1,358 (10.47) | 279 (10.55) | 339 (11.13) | 0.003 | 0.021 | 0.019 |
| Osteoporosis | 352 (2.71) | 53 (2) | 54 (1.77) | 0.047 | 0.064 | 0.017 |
| Osteoporosis fracture | 282 (2.17) | 48 (1.81) | 39 (1.28) | 0.026 | 0.069 | 0.043 |
| Other connective tissue inflammatory diseases | 250 (1.93) | 45 (1.7) | 44 (1.44) | 0.017 | 0.038 | 0.021 |
| Other non-inflammatory diseases | 266 (2.05) | 87 (3.29) | 114 (3.74) | 0.077 | 0.101 | 0.025 |
| Pain in joint | 1,408 (10.85) | 295 (11.15) | 317 (10.41) | 0.01 | 0.015 | 0.024 |
| Peripheral neuropathy | 342 (2.64) | 65 (2.46) | 103 (3.38) | 0.011 | 0.044 | 0.055 |
| Spondyloarthropathies | 132 (1.02) | 8 (0.3) | 13 (0.43) | 0.088 | 0.07 | 0.021 |
| Tendinitis | 534 (4.12) | 126 (4.76) | 174 (5.71) | 0.031 | 0.074 | 0.043 |
| Tendinitis lower extremities | 919 (7.09) | 251 (9.49) | 270 (8.86) | 0.087 | 0.066 | 0.022 |
| Tendinitis upper extremities | 2,038 (15.71) | 476 (18) | 586 (19.24) | 0.061 | 0.093 | 0.032 |
| Treatment at discharge |  |  |  |  |  |  |
| Analgesic 1st level | 4,828 (37.22) | 1,022 (38.64) | 1,138 (37.36) | 0.029 | 0.003 | 0.026 |
| Analgesic 2nd and 3rd level | 1,178 (9.08) | 241 (9.11) | 290 (9.52) | 0.001 | 0.015 | 0.014 |
| Antidepressant | 168 (1.3) | 49 (1.85) | 38 (1.25) | 0.045 | 0.004 | 0.049 |
| Antiosteoporotic | 452 (3.48) | 52 (1.97) | 44 (1.44) | 0.093 | 0.132 | 0.04 |
| Benzodiazepine | 1017 (7.84) | 161 (6.09) | 175 (5.75) | 0.069 | 0.083 | 0.014 |
| Calcium and vitamin D | 951 (7.33) | 201 (7.6) | 200 (6.57) | 0.01 | 0.03 | 0.04 |
| Colchicine | 204 (1.57) | 36 (1.36) | 41 (1.35) | 0.018 | 0.019 | 0.001 |
| Corticosteroid | 622 (4.8) | 86 (3.25) | 127 (4.17) | 0.079 | 0.03 | 0.049 |
| Gabapentin | 235 (1.81) | 70 (2.65) | 80 (2.63) | 0.057 | 0.055 | 0.001 |
| Gastric protector | 1,966 (15.16) | 314 (11.87) | 266 (8.73) | 0.096 | 0.199 | 0.103 |
| Lowering uric acid drugs | 133 (1.03) | 35 (1.32) | 23 (0.76) | 0.028 | 0.029 | 0.056 |
| NSAIDs | 4,240 (32.69) | 842 (31.83) | 904 (29.68) | 0.018 | 0.065 | 0.047 |
| NSAIDs hard | 158 (1.22) | 17 (0.64) | 13 (0.43) | 0.06 | 0.088 | 0.03 |
| Other DMARDs | 261 (2.01) | 14 (0.53) | 22 (0.72) | 0.133 | 0.111 | 0.024 |
| Other drugs | 208 (1.6) | 31 (1.17) | 63 (2.07) | 0.037 | 0.035 | 0.071 |
| SYSADOA | 305 (2.35) | 49 (1.85) | 56 (1.84) | 0.035 | 0.036 | 0.001 |
| Diagnosis registered in the last 90 days before discharge |  |  |  |  |  |  |
| Axial neuropathy | 396 (3.05) | 82 (3.1) | 95 (3.12) | 0.003 | 0.004 | 0.001 |
| Back pain | 2,204 (16.99) | 469 (17.73) | 499 (16.38) | 0.02 | 0.016 | 0.036 |
| Chronic polyarthritis | 348 (2.68) | 33 (1.25) | 43 (1.41) | 0.104 | 0.09 | 0.014 |
| Crystal arthropathy | 378 (2.91) | 98 (3.71) | 101 (3.32) | 0.044 | 0.023 | 0.021 |
| Fibromyalgia | 182 (1.4) | 37 (1.4) | 36 (1.18) | 0 | 0.02 | 0.019 |
| Gout | 282 (2.17) | 51 (1.93) | 49 (1.61) | 0.017 | 0.042 | 0.024 |
| Muscle disorders | 569 (4.39) | 121 (4.57) | 119 (3.91) | 0.009 | 0.024 | 0.033 |
| Neck pain | 914 (7.05) | 152 (5.75) | 154 (5.06) | 0.053 | 0.084 | 0.031 |
| No diagnoses | 1,616 (12.46) | 318 (12.02) | 359 (11.79) | 0.013 | 0.021 | 0.007 |
| Generalized or Unspecified Osteoarthritis | 1,004 (7.74) | 158 (5.97) | 221 (7.26) | 0.07 | 0.018 | 0.052 |
| Osteoarthritis of first carpometacarpal joints | 338 (2.61) | 63 (2.38) | 81 (2.66) | 0.014 | 0.003 | 0.018 |
| Osteoarthritis of hand | 718 (5.54) | 133 (5.03) | 146 (4.79) | 0.023 | 0.034 | 0.011 |
| Osteoarthritis of hip | 263 (2.03) | 57 (2.16) | 69 (2.27) | 0.009 | 0.016 | 0.007 |
| Osteoarthritis of knee | 1378 (10.62) | 289 (10.93) | 355 (11.65) | 0.01 | 0.033 | 0.023 |
| Osteoporosis | 363 (2.8) | 56 (2.12) | 55 (1.81) | 0.044 | 0.066 | 0.022 |
| Osteoporosis fracture | 286 (2.2) | 51 (1.93) | 40 (1.31) | 0.019 | 0.068 | 0.049 |
| Other connective tissue inflammatory diseases | 256 (1.97) | 46 (1.74) | 50 (1.64) | 0.017 | 0.025 | 0.008 |
| Other non-inflammatory diseases | 287 (2.21) | 100 (3.78) | 123 (4.04) | 0.092 | 0.105 | 0.013 |
| Pain in joint | 1,501 (11.57) | 338 (12.78) | 357 (11.72) | 0.037 | 0.005 | 0.032 |
| Peripheral neuropathy | 360 (2.78) | 77 (2.91) | 112 (3.68) | 0.008 | 0.051 | 0.043 |
| Spondyloarthropathies | 144 (1.11) | 8 (0.3) | 17 (0.56) | 0.097 | 0.061 | 0.039 |
| Tendinitis | 566 (4.36) | 140 (5.29) | 185 (6.07) | 0.043 | 0.077 | 0.034 |
| Tendinitis lower extremities | 964 (7.43) | 266 (10.06) | 287 (9.42) | 0.093 | 0.072 | 0.021 |
| Tendinitis upper extremities | 2,085 (16.07) | 487 (18.41) | 602 (19.76) | 0.062 | 0.096 | 0.034 |
| Treatments registered in the last 90 days before discharge |  |  |  |  |  |  |
| Analgesic 1st level | 4,927 (37.98) | 1,046 (39.55) | 1,176 (38.61) | 0.032 | 0.013 | 0.019 |
| Analgesic 2nd and 3rd level | 1,232 (9.5) | 258 (9.75) | 305 (10.01) | 0.009 | 0.017 | 0.009 |
| Antidepressant | 171 (1.32) | 51 (1.93) | 41 (1.35) | 0.048 | 0.002 | 0.046 |
| Antiosteoporotic | 458 (3.53) | 55 (2.08) | 46 (1.51) | 0.088 | 0.129 | 0.043 |
| Benzodiazepine | 1,076 (8.3) | 176 (6.65) | 194 (6.37) | 0.062 | 0.074 | 0.012 |
| Calcium and vitamin D | 968 (7.46) | 207 (7.83) | 207 (6.8) | 0.014 | 0.026 | 0.04 |
| Colchicine | 218 (1.68) | 41 (1.55) | 47 (1.54) | 0.01 | 0.011 | 0.001 |
| Corticosteroid | 741 (5.71) | 119 (4.5) | 171 (5.61) | 0.055 | 0.004 | 0.051 |
| Gabapentin | 254 (1.96) | 75 (2.84) | 90 (2.95) | 0.057 | 0.064 | 0.007 |
| Gastric protector | 2,087 (16.09) | 351 (13.27) | 314 (10.31) | 0.08 | 0.171 | 0.092 |
| Lowering uric acid drugs | 133 (1.03) | 35 (1.32) | 23 (0.76) | 0.028 | 0.029 | 0.056 |
| NSAIDs | 4,458 (34.37) | 900 (34.03) | 989 (32.47) | 0.007 | 0.04 | 0.033 |
| NSAIDs hard | 170 (1.31) | 20 (0.76) | 15 (0.49) | 0.055 | 0.087 | 0.033 |
| Other DMARDs | 272 (2.1) | 14 (0.53) | 24 (0.79) | 0.138 | 0.11 | 0.032 |
| Other drugs | 236 (1.82) | 40 (1.51) | 72 (2.36) | 0.024 | 0.038 | 0.062 |
| SYSADOA | 306 (2.36) | 50 (1.89) | 57 (1.87) | 0.033 | 0.034 | 0.001 |
| Diagnosis registered in the last 182 days before discharge |  |  |  |  |  |  |
| Axial neuropathy | 404 (3.11) | 88 (3.33) | 96 (3.15) | 0.012 | 0.002 | 0.01 |
| Back pain | 2,229 (17.18) | 473 (17.88) | 506 (16.61) | 0.018 | 0.015 | 0.034 |
| Chronic polyarthritis | 360 (2.78) | 34 (1.29) | 46 (1.51) | 0.106 | 0.087 | 0.019 |
| Crystal arthropathy | 385 (2.97) | 100 (3.78) | 109 (3.58) | 0.045 | 0.034 | 0.011 |
| Fibromyalgia | 185 (1.43) | 37 (1.4) | 36 (1.18) | 0.002 | 0.022 | 0.019 |
| Gout | 283 (2.18) | 51 (1.93) | 49 (1.61) | 0.018 | 0.042 | 0.024 |
| Muscle disorders | 583 (4.49) | 123 (4.65) | 124 (4.07) | 0.007 | 0.021 | 0.028 |
| Neck pain | 927 (7.15) | 157 (5.94) | 157 (5.15) | 0.049 | 0.083 | 0.034 |
| No diagnoses | 1,693 (13.05) | 324 (12.25) | 369 (12.11) | 0.024 | 0.028 | 0.004 |
| Generalized or Unspecified Osteoarthritis | 1015 (7.83) | 159 (6.01) | 224 (7.35) | 0.072 | 0.018 | 0.054 |
| Osteoarthritis of first carpometacarpal joints | 341 (2.63) | 63 (2.38) | 83 (2.72) | 0.016 | 0.006 | 0.022 |
| Osteoarthritis of hand | 727 (5.6) | 133 (5.03) | 146 (4.79) | 0.026 | 0.037 | 0.011 |
| Osteoarthritis of hip | 270 (2.08) | 58 (2.19) | 69 (2.27) | 0.008 | 0.013 | 0.005 |
| Osteoarthritis of knee | 1,387 (10.69) | 290 (10.96) | 358 (11.75) | 0.009 | 0.034 | 0.025 |
| Osteoporosis | 366 (2.82) | 56 (2.12) | 55 (1.81) | 0.045 | 0.068 | 0.022 |
| Osteoporosis fracture | 288 (2.22) | 51 (1.93) | 42 (1.38) | 0.021 | 0.063 | 0.043 |
| Other connective tissue inflammatory diseases | 265 (2.04) | 46 (1.74) | 51 (1.67) | 0.022 | 0.027 | 0.005 |
| Other non-inflammatory diseases | 290 (2.24) | 103 (3.89) | 125 (4.1) | 0.096 | 0.107 | 0.011 |
| Pain in joint | 1,533 (11.82) | 355 (13.42) | 370 (12.15) | 0.048 | 0.01 | 0.038 |
| Peripheral neuropathy | 373 (2.88) | 81 (3.06) | 116 (3.81) | 0.011 | 0.052 | 0.041 |
| Spondyloarthropathies | 152 (1.17) | 9 (0.34) | 17 (0.56) | 0.096 | 0.066 | 0.033 |
| Tendinitis | 577 (4.45) | 141 (5.33) | 190 (6.24) | 0.041 | 0.08 | 0.039 |
| Tendinitis lower extremities | 993 (7.66) | 275 (10.4) | 295 (9.68) | 0.096 | 0.072 | 0.024 |
| Tendinitis upper extremities | 2102 (16.21) | 499 (18.87) | 608 (19.96) | 0.07 | 0.098 | 0.028 |
| Treatments registered in the last 182 days before discharge |  |  |  |  |  |  |
| Analgesic 1st level | 4965 (38.28) | 1,061 (40.11) | 1,183 (38.84) | 0.038 | 0.012 | 0.026 |
| Analgesic 2nd and 3rd level | 1,267 (9.77) | 261 (9.87) | 310 (10.18) | 0.003 | 0.014 | 0.01 |
| Antidepressant | 176 (1.36) | 56 (2.12) | 43 (1.41) | 0.058 | 0.005 | 0.054 |
| Antiosteoporotic | 466 (3.59) | 56 (2.12) | 46 (1.51) | 0.089 | 0.132 | 0.046 |
| Benzodiazepine | 1,109 (8.55) | 183 (6.92) | 199 (6.53) | 0.061 | 0.076 | 0.015 |
| Calcium and vitamin D | 989 (7.62) | 210 (7.94) | 207 (6.8) | 0.012 | 0.032 | 0.044 |
| Colchicine | 232 (1.79) | 46 (1.74) | 51 (1.67) | 0.004 | 0.009 | 0.005 |
| Corticosteroid | 795 (6.13) | 133 (5.03) | 185 (6.07) | 0.048 | 0.002 | 0.046 |
| Gabapentin | 267 (2.06) | 80 (3.02) | 92 (3.02) | 0.061 | 0.061 | 0 |
| Gastric protector | 2,130 (16.42) | 365 (13.8) | 323 (10.6) | 0.073 | 0.171 | 0.098 |
| Lowering uric acid drugs | 135 (1.04) | 35 (1.32) | 23 (0.76) | 0.026 | 0.03 | 0.056 |
| NSAIDs | 4,531 (34.93) | 931 (35.2) | 1,011 (33.19) | 0.006 | 0.037 | 0.042 |
| NSAIDs hard | 180 (1.39) | 21 (0.79) | 15 (0.49) | 0.057 | 0.093 | 0.038 |
| Other DMARDs | 279 (2.15) | 18 (0.68) | 26 (0.85) | 0.125 | 0.107 | 0.02 |
| Other drugs | 249 (1.92) | 44 (1.66) | 74 (2.43) | 0.019 | 0.035 | 0.054 |
| SYSADOA | 310 (2.39) | 50 (1.89) | 58 (1.9) | 0.035 | 0.034 | 0.001 |
| Diagnosis at first visit of episode |  |  |  |  |  |  |
| Axial neuropathy | 374 (2.88) | 73 (2.76) | 88 (2.89) | 0.007 | 0 | 0.008 |
| Back pain | 2,051 (15.81) | 439 (16.6) | 473 (15.53) | 0.021 | 0.008 | 0.029 |
| Chronic polyarthritis | 326 (2.51) | 37 (1.4) | 52 (1.71) | 0.081 | 0.056 | 0.025 |
| Crystal arthropathy | 353 (2.72) | 92 (3.48) | 100 (3.28) | 0.044 | 0.033 | 0.011 |
| Fibromyalgia | 166 (1.28) | 28 (1.06) | 31 (1.02) | 0.021 | 0.025 | 0.004 |
| Gout | 269 (2.07) | 46 (1.74) | 48 (1.58) | 0.024 | 0.037 | 0.013 |
| Muscle disorders | 533 (4.11) | 112 (4.23) | 120 (3.94) | 0.006 | 0.009 | 0.015 |
| Neck pain | 879 (6.78) | 145 (5.48) | 155 (5.09) | 0.054 | 0.072 | 0.018 |
| No diagnoses | 1,656 (12.77) | 318 (12.02) | 362 (11.88) | 0.023 | 0.027 | 0.004 |
| Generalized or Unspecified Osteoarthritis | 907 (6.99) | 144 (5.44) | 195 (6.4) | 0.064 | 0.024 | 0.041 |
| Osteoarthritis of first carpometacarpal joints | 307 (2.37) | 55 (2.08) | 74 (2.43) | 0.019 | 0.004 | 0.024 |
| Osteoarthritis of hand | 691 (5.33) | 123 (4.65) | 137 (4.5) | 0.031 | 0.038 | 0.007 |
| Osteoarthritis of hip | 229 (1.77) | 46 (1.74) | 60 (1.97) | 0.002 | 0.015 | 0.017 |
| Osteoarthritis of knee | 1,254 (9.67) | 253 (9.57) | 308 (10.11) | 0.003 | 0.015 | 0.018 |
| Osteoporosis | 333 (2.57) | 48 (1.81) | 52 (1.71) | 0.051 | 0.059 | 0.008 |
| Osteoporosis fracture | 234 (1.8) | 35 (1.32) | 40 (1.31) | 0.039 | 0.04 | 0.001 |
| Other connective tissue inflammatory diseases | 244 (1.88) | 46 (1.74) | 45 (1.48) | 0.011 | 0.031 | 0.021 |
| Other non-inflammatory diseases | 255 (1.97) | 77 (2.91) | 107 (3.51) | 0.061 | 0.095 | 0.034 |
| Pain in joint | 1,442 (11.12) | 324 (12.25) | 343 (11.26) | 0.035 | 0.005 | 0.031 |
| Peripheral neuropathy | 334 (2.57) | 68 (2.57) | 104 (3.41) | 0 | 0.049 | 0.05 |
| Spondyloarthropathies | 126 (0.97) | 9 (0.34) | 12 (0.39) | 0.078 | 0.07 | 0.009 |
| Tendinitis | 531 (4.09) | 125 (4.73) | 155 (5.09) | 0.031 | 0.048 | 0.017 |
| Tendinitis lower extremities | 898 (6.92) | 249 (9.41) | 265 (8.7) | 0.091 | 0.066 | 0.025 |
| Tendinitis upper extremities | 2,015 (15.53) | 466 (17.62) | 565 (18.55) | 0.056 | 0.08 | 0.024 |
| Treatment at first visit of episode |  |  |  |  |  |  |
| Analgesic 1st level | 4,571 (35.24) | 937 (35.43) | 1,071 (35.16) | 0.004 | 0.002 | 0.006 |
| Analgesic 2nd and 3rd level | 1,109 (8.55) | 205 (7.75) | 244 (8.01) | 0.029 | 0.02 | 0.01 |
| Antidepressant | 155 (1.19) | 39 (1.47) | 38 (1.25) | 0.024 | 0.005 | 0.02 |
| Antiosteoporotic | 364 (2.81) | 41 (1.55) | 36 (1.18) | 0.086 | 0.116 | 0.032 |
| Benzodiazepine | 1,035 (7.98) | 171 (6.47) | 187 (6.14) | 0.059 | 0.072 | 0.013 |
| Calcium and vitamin D | 786 (6.06) | 137 (5.18) | 132 (4.33) | 0.038 | 0.078 | 0.04 |
| Colchicine | 193 (1.49) | 42 (1.59) | 40 (1.31) | 0.008 | 0.015 | 0.023 |
| Corticosteroid | 723 (5.57) | 123 (4.65) | 162 (5.32) | 0.042 | 0.011 | 0.031 |
| Gabapentin | 223 (1.72) | 58 (2.19) | 69 (2.27) | 0.034 | 0.039 | 0.005 |
| Gastric protector | 2,009 (15.49) | 338 (12.78) | 315 (10.34) | 0.078 | 0.154 | 0.076 |
| Lowering uric acid drugs | 102 (0.79) | 21 (0.79) | 11 (0.36) | 0.001 | 0.056 | 0.057 |
| NSAIDs | 4,264 (32.87) | 846 (31.98) | 932 (30.6) | 0.019 | 0.049 | 0.03 |
| NSAIDs hard | 164 (1.26) | 19 (0.72) | 18 (0.59) | 0.055 | 0.07 | 0.016 |
| Other DMARDs | 217 (1.67) | 637 (29.26) | 12 (0.39) | 0.119 | 0.127 | 0.009 |
| Other drugs | 181 (1.4) | 12 (0.45) | 52 (1.71) | 0.042 | 0.025 | 0.067 |
| SYSADOA | 264 (2.04) | 25 (0.95) | 47 (1.54) | 0.022 | 0.037 | 0.015 |
| Comorbidities at discharge |  |  |  |  |  |  |
| Alcoholic habit | 218 (1.68) | 44 (1.66) | 47 (1.54) | 0.001 | 0.011 | 0.01 |
| Allergy | 214 (1.65) | 58 (2.19) | 71 (2.33) | 0.04 | 0.049 | 0.009 |
| Allergy medication | 109 (0.84) | 39 (1.47) | 32 (1.05) | 0.059 | 0.022 | 0.038 |
| Alpha-blocker | 99 (0.76) | 20 (0.76) | 26 (0.85) | 0.001 | 0.01 | 0.011 |
| Ambiental allergy | 198 (1.53) | 55 (2.08) | 67 (2.2) | 0.042 | 0.05 | 0.008 |
| Anemia | 164 (1.26) | 30 (1.13) | 30 (0.98) | 0.012 | 0.027 | 0.015 |
| Angiotensin II receptor blocker | 914 (7.05) | 179 (6.77) | 241 (7.91) | 0.011 | 0.033 | 0.044 |
| Angiotensin-converting-enzyme inhibitor | 987 (7.61) | 224 (8.47) | 269 (8.83) | 0.032 | 0.044 | 0.013 |
| Antiaggregant | 836 (6.45) | 143 (5.41) | 176 (5.78) | 0.044 | 0.028 | 0.016 |
| Anticoagulant | 402 (3.1) | 83 (3.14) | 103 (3.38) | 0.002 | 0.016 | 0.014 |
| Antidepressant | 838 (6.46) | 193 (7.3) | 226 (7.42) | 0.033 | 0.038 | 0.005 |
| Antiepileptics | 110 (0.85) | 27 (1.02) | 33 (1.08) | 0.018 | 0.024 | 0.006 |
| Anxiety | 238 (1.83) | 59 (2.23) | 60 (1.97) | 0.028 | 0.01 | 0.018 |
| Anxiety medication | 946 (7.29) | 202 (7.64) | 200 (6.57) | 0.013 | 0.029 | 0.042 |
| Appendicitis | 360 (2.78) | 63 (2.38) | 78 (2.56) | 0.025 | 0.013 | 0.012 |
| Articular prosthesis | 389 (3) | 81 (3.06) | 112 (3.68) | 0.004 | 0.038 | 0.034 |
| Articular surgery no prosthesis | 69 (0.53) | 14 (0.53) | 20 (0.66) | 0 | 0.016 | 0.017 |
| Asthma | 298 (2.3) | 70 (2.65) | 62 (2.04) | 0.022 | 0.018 | 0.04 |
| Atrial fibrillation | 267 (2.06) | 54 (2.04) | 70 (2.3) | 0.001 | 0.016 | 0.018 |
| Axial neuropathy | 88 (0.68) | 19 (0.72) | 19 (0.62) | 0.005 | 0.007 | 0.012 |
| Back pain | 333 (2.57) | 58 (2.19) | 79 (2.59) | 0.025 | 0.002 | 0.026 |
| Benign breast tumor | 126 (0.97) | 20 (0.76) | 28 (0.92) | 0.023 | 0.005 | 0.018 |
| Benign prostatic hyperplasia medication | 197 (1.52) | 43 (1.63) | 52 (1.71) | 0.009 | 0.015 | 0.006 |
| Benign prostatic hypertrophy | 278 (2.14) | 67 (2.53) | 68 (2.23) | 0.026 | 0.006 | 0.02 |
| Benzodiazepine | 921 (7.1) | 197 (7.45) | 197 (6.47) | 0.013 | 0.025 | 0.039 |
| Beta-blocker | 575 (4.43) | 127 (4.8) | 143 (4.69) | 0.018 | 0.013 | 0.005 |
| Bisphosphonates | 385 (2.97) | 63 (2.38) | 57 (1.87) | 0.036 | 0.071 | 0.035 |
| Breast cancer | 172 (1.33) | 65 (2.46) | 38 (1.25) | 0.083 | 0.007 | 0.09 |
| Bronchodilator | 246 (1.9) | 54 (2.04) | 64 (2.1) | 0.01 | 0.015 | 0.004 |
| Calcium | 689 (5.31) | 146 (5.52) | 173 (5.68) | 0.009 | 0.016 | 0.007 |
| Calcium antagonist | 600 (4.63) | 126 (4.76) | 175 (5.75) | 0.007 | 0.051 | 0.044 |
| Cancer | 357 (2.75) | 62 (2.34) | 85 (2.79) | 0.026 | 0.002 | 0.028 |
| Cancer medication | 168 (1.3) | 46 (1.74) | 49 (1.61) | 0.036 | 0.026 | 0.01 |
| Cataract | 260 (2) | 49 (1.85) | 61 (2) | 0.011 | 0 | 0.011 |
| Central nervous system diseases | 196 (1.51) | 52 (1.97) | 53 (1.74) | 0.035 | 0.018 | 0.017 |
| Cerebrovascular disease | 190 (1.46) | 28 (1.06) | 41 (1.35) | 0.036 | 0.01 | 0.026 |
| Cervicalgia | 119 (0.92) | 22 (0.83) | 37 (1.21) | 0.009 | 0.029 | 0.038 |
| Cholecystectomy | 358 (2.76) | 65 (2.46) | 76 (2.5) | 0.019 | 0.017 | 0.002 |
| Chronic obstructive pulmonary disease | 229 (1.77) | 43 (1.63) | 54 (1.77) | 0.011 | 0.001 | 0.011 |
| Cognitive impairment | 74 (0.57) | 17 (0.64) | 23 (0.76) | 0.009 | 0.023 | 0.013 |
| Colon cancer | 80 (0.62) | 18 (0.68) | 13 (0.43) | 0.008 | 0.026 | 0.034 |
| Colon diverticulitis | 80 (0.62) | 26 (0.98) | 21 (0.69) | 0.041 | 0.009 | 0.032 |
| Colon hemorrhoid | 81 (0.62) | 12 (0.45) | 17 (0.56) | 0.023 | 0.009 | 0.015 |
| Colon surgery | 109 (0.84) | 24 (0.91) | 30 (0.98) | 0.007 | 0.015 | 0.008 |
| Column surgery | 224 (1.73) | 41 (1.55) | 68 (2.23) | 0.014 | 0.036 | 0.05 |
| Connective tissue benign tumor | 153 (1.18) | 34 (1.29) | 45 (1.48) | 0.01 | 0.026 | 0.016 |
| Constipation | 132 (1.02) | 27 (1.02) | 20 (0.66) | 0 | 0.04 | 0.04 |
| Contraception | 97 (0.75) | 20 (0.76) | 15 (0.49) | 0.001 | 0.033 | 0.033 |
| Cystocele | 83 (0.64) | 18 (0.68) | 27 (0.89) | 0.005 | 0.028 | 0.023 |
| Depression | 653 (5.03) | 139 (5.26) | 151 (4.96) | 0.01 | 0.004 | 0.014 |
| Diabetes mellitus | 1089 (8.4) | 238 (9) | 275 (9.03) | 0.021 | 0.022 | 0.001 |
| Digoxin | 83 (0.64) | 13 (0.49) | 9 (0.3) | 0.02 | 0.05 | 0.031 |
| Disease modifying antirheumatic drugs | 61 (0.47) | 22 (0.83) | 33 (1.08) | 0.045 | 0.07 | 0.026 |
| Diuretic | 1211 (9.34) | 245 (9.26) | 282 (9.26) | 0.003 | 0.003 | 0 |
| Dyslipidemia | 2769 (21.35) | 605 (22.87) | 719 (23.6) | 0.037 | 0.054 | 0.017 |
| Fall | 74 (0.57) | 25 (0.95) | 16 (0.53) | 0.043 | 0.006 | 0.049 |
| Family history | 80 (0.62) | 21 (0.79) | 20 (0.66) | 0.021 | 0.005 | 0.016 |
| Feet diseases | 255 (1.97) | 55 (2.08) | 72 (2.36) | 0.008 | 0.027 | 0.019 |
| Gabapentin | 190 (1.46) | 48 (1.81) | 53 (1.74) | 0.028 | 0.022 | 0.006 |
| Gastritis | 133 (1.03) | 27 (1.02) | 37 (1.21) | 0 | 0.018 | 0.018 |
| Glaucoma | 149 (1.15) | 29 (1.1) | 23 (0.76) | 0.005 | 0.041 | 0.036 |
| Gout | 87 (0.67) | 15 (0.57) | 25 (0.82) | 0.013 | 0.017 | 0.031 |
| Hand diseases | 158 (1.22) | 39 (1.47) | 31 (1.02) | 0.022 | 0.019 | 0.041 |
| Helicobacter pylori infection | 75 (0.58) | 22 (0.83) | 22 (0.72) | 0.03 | 0.018 | 0.012 |
| Hematopoietic cancer | 73 (0.56) | 22 (0.83) | 22 (0.72) | 0.032 | 0.02 | 0.012 |
| Hiatal hernia | 350 (2.7) | 71 (2.68) | 65 (2.13) | 0.001 | 0.037 | 0.036 |
| Hip prosthesis | 86 (0.66) | 22 (0.83) | 32 (1.05) | 0.02 | 0.042 | 0.023 |
| Hyperglycemia | 138 (1.06) | 30 (1.13) | 16 (0.53) | 0.007 | 0.061 | 0.067 |
| Hypertension | 3,376 (26.03) | 665 (25.14) | 836 (27.45) | 0.02 | 0.032 | 0.052 |
| Hyperthyroidism | 78 (0.6) | 16 (0.6) | 10 (0.33) | 0 | 0.04 | 0.041 |
| Hyperuricemia | 166 (1.28) | 43 (1.63) | 32 (1.05) | 0.029 | 0.021 | 0.05 |
| Hypothyroidism | 772 (5.95) | 180 (6.81) | 209 (6.86) | 0.035 | 0.037 | 0.002 |
| Hysterectomy surgery | 378 (2.91) | 66 (2.5) | 86 (2.82) | 0.026 | 0.005 | 0.02 |
| Inhaled corticosteroid | 217 (1.67) | 48 (1.81) | 59 (1.94) | 0.011 | 0.02 | 0.009 |
| Insomnia | 76 (0.59) | 11 (0.42) | 16 (0.53) | 0.024 | 0.008 | 0.016 |
| Insulin | 246 (1.9) | 50 (1.89) | 71 (2.33) | 0 | 0.03 | 0.031 |
| Internal derangement of knee | 192 (1.48) | 38 (1.44) | 47 (1.54) | 0.004 | 0.005 | 0.009 |
| Iron | 106 (0.82) | 25 (0.95) | 37 (1.21) | 0.014 | 0.04 | 0.026 |
| Irritable colon syndrome | 97 (0.75) | 22 (0.83) | 12 (0.39) | 0.009 | 0.047 | 0.056 |
| Ischemic heart disease | 375 (2.89) | 63 (2.38) | 81 (2.66) | 0.032 | 0.014 | 0.018 |
| Kidney failure | 158 (1.22) | 38 (1.44) | 39 (1.28) | 0.019 | 0.006 | 0.014 |
| Knee arthrosis | 93 (0.72) | 21 (0.79) | 27 (0.89) | 0.009 | 0.019 | 0.01 |
| Knee surgery non prosthesis | 227 (1.75) | 49 (1.85) | 56 (1.84) | 0.008 | 0.007 | 0.001 |
| Knee surgery prosthesis | 133 (1.03) | 42 (1.59) | 43 (1.41) | 0.05 | 0.035 | 0.014 |
| Lowering uric acid drugs | 185 (1.43) | 59 (2.23) | 51 (1.67) | 0.06 | 0.02 | 0.04 |
| Macrocytosis | 69 (0.53) | 20 (0.76) | 15 (0.49) | 0.028 | 0.006 | 0.033 |
| Metamizole | 92 (0.71) | 28 (1.06) | 32 (1.05) | 0.037 | 0.037 | 0.001 |
| Migraine | 189 (1.46) | 35 (1.32) | 44 (1.44) | 0.011 | 0.001 | 0.01 |
| Non osteoporotic fracture | 209 (1.61) | 52 (1.97) | 45 (1.48) | 0.027 | 0.011 | 0.038 |
| NSAIDs | 227 (1.75) | 38 (1.44) | 28 (0.92) | 0.025 | 0.072 | 0.048 |
| Obesity | 484 (3.73) | 91 (3.44) | 140 (4.6) | 0.016 | 0.043 | 0.059 |
| Ocular hypertension medication | 144 (1.11) | 26 (0.98) | 29 (0.95) | 0.012 | 0.016 | 0.003 |
| Oral anticoagulant | 399 (3.08) | 81 (3.06) | 101 (3.32) | 0.001 | 0.014 | 0.014 |
| Oral antidiabetics | 777 (5.99) | 194 (7.33) | 203 (6.66) | 0.054 | 0.028 | 0.026 |
| Osteoporosis | 412 (3.18) | 92 (3.48) | 89 (2.92) | 0.017 | 0.015 | 0.032 |
| Osteoporotic fracture | 203 (1.57) | 54 (2.04) | 49 (1.61) | 0.036 | 0.003 | 0.032 |
| Other antihypertensives | 379 (2.92) | 92 (3.48) | 86 (2.82) | 0.032 | 0.006 | 0.037 |
| Other arrhythmias | 448 (3.45) | 86 (3.25) | 110 (3.61) | 0.011 | 0.009 | 0.02 |
| Other articular surgeries | 374 (2.88) | 77 (2.91) | 104 (3.41) | 0.002 | 0.03 | 0.029 |
| Other benign tumors | 138 (1.06) | 30 (1.13) | 41 (1.35) | 0.007 | 0.026 | 0.019 |
| Other biliary diseases | 425 (3.28) | 75 (2.84) | 91 (2.99) | 0.026 | 0.017 | 0.009 |
| Other colon diseases | 165 (1.27) | 27 (1.02) | 46 (1.51) | 0.024 | 0.02 | 0.044 |
| Other dermatological diseases | 188 (1.45) | 35 (1.32) | 51 (1.67) | 0.011 | 0.018 | 0.029 |
| Other digestive diseases | 336 (2.59) | 63 (2.38) | 91 (2.99) | 0.013 | 0.024 | 0.037 |
| Other ear diseases | 160 (1.23) | 33 (1.25) | 35 (1.15) | 0.001 | 0.008 | 0.009 |
| Other endocrine diseases | 190 (1.46) | 40 (1.51) | 51 (1.67) | 0.004 | 0.017 | 0.013 |
| Other esophagus diseases | 284 (2.19) | 65 (2.46) | 71 (2.33) | 0.018 | 0.01 | 0.008 |
| Other eyes diseases | 166 (1.28) | 50 (1.89) | 40 (1.31) | 0.049 | 0.003 | 0.046 |
| Other heart diseases | 93 (0.72) | 17 (0.64) | 19 (0.62) | 0.009 | 0.011 | 0.002 |
| Other hematologic diseases | 216 (1.67) | 45 (1.7) | 44 (1.44) | 0.003 | 0.018 | 0.021 |
| Other hip diseases | 83 (0.64) | 14 (0.53) | 17 (0.56) | 0.015 | 0.011 | 0.004 |
| Other infection | 245 (1.89) | 64 (2.42) | 49 (1.61) | 0.037 | 0.021 | 0.058 |
| Other knee disease | 80 (0.62) | 13 (0.49) | 16 (0.53) | 0.017 | 0.012 | 0.005 |
| Other liver disease | 164 (1.26) | 30 (1.13) | 36 (1.18) | 0.012 | 0.008 | 0.004 |
| Other musculoskeletal infection | 231 (1.78) | 71 (2.68) | 62 (2.04) | 0.061 | 0.019 | 0.043 |
| Other neurological diseases | 86 (0.66) | 22 (0.83) | 19 (0.62) | 0.02 | 0.005 | 0.024 |
| Other otorhinolaryngology diseases | 219 (1.69) | 46 (1.74) | 53 (1.74) | 0.004 | 0.004 | 0 |
| Other ovary diseases | 126 (0.97) | 28 (1.06) | 24 (0.79) | 0.009 | 0.02 | 0.028 |
| Other psychiatric conditions | 118 (0.91) | 26 (0.98) | 19 (0.62) | 0.008 | 0.033 | 0.04 |
| Other respiratory diseases | 81 (0.62) | 24 (0.91) | 12 (0.39) | 0.032 | 0.032 | 0.064 |
| Other spine diseases | 157 (1.21) | 26 (0.98) | 35 (1.15) | 0.022 | 0.006 | 0.016 |
| Other urological diseases | 79 (0.61) | 16 (0.6) | 26 (0.85) | 0.001 | 0.029 | 0.029 |
| Other uterus diseases | 327 (2.52) | 59 (2.23) | 75 (2.46) | 0.019 | 0.004 | 0.015 |
| Other valve diseases | 146 (1.13) | 32 (1.21) | 35 (1.15) | 0.008 | 0.002 | 0.006 |
| Other virus infection | 291 (2.24) | 65 (2.46) | 62 (2.04) | 0.014 | 0.014 | 0.028 |
| Paracetamol | 320 (2.47) | 67 (2.53) | 99 (3.25) | 0.004 | 0.047 | 0.043 |
| Parkinson's disease | 72 (0.56) | 11 (0.42) | 19 (0.62) | 0.02 | 0.009 | 0.029 |
| Peptic ulcer | 204 (1.57) | 39 (1.47) | 27 (0.89) | 0.008 | 0.062 | 0.054 |
| Peripheral artery disease | 83 (0.64) | 21 (0.79) | 30 (0.98) | 0.018 | 0.038 | 0.02 |
| Peripheral nervous system diseases | 289 (2.23) | 65 (2.46) | 84 (2.76) | 0.015 | 0.034 | 0.019 |
| Pregnancy | 246 (1.9) | 44 (1.66) | 57 (1.87) | 0.018 | 0.002 | 0.016 |
| Prostate cancer | 79 (0.61) | 17 (0.64) | 21 (0.69) | 0.004 | 0.01 | 0.006 |
| Proton-pump inhibitor medication | 1,087 (8.38) | 241 (9.11) | 270 (8.86) | 0.026 | 0.017 | 0.009 |
| Psoriasis | 138 (1.06) | 33 (1.25) | 55 (1.81) | 0.017 | 0.062 | 0.046 |
| Pulmonary embolism or deep vein thrombosis | 90 (0.69) | 21 (0.79) | 23 (0.76) | 0.012 | 0.007 | 0.004 |
| Salpingectomy and oophorectomy | 211 (1.63) | 42 (1.59) | 30 (0.98) | 0.003 | 0.057 | 0.054 |
| Second and third analgesia level | 236 (1.82) | 55 (2.08) | 77 (2.53) | 0.019 | 0.049 | 0.03 |
| Shoulder diseases | 112 (0.86) | 21 (0.79) | 43 (1.41) | 0.008 | 0.052 | 0.059 |
| Sleep apnea | 68 (0.52) | 13 (0.49) | 25 (0.82) | 0.005 | 0.036 | 0.041 |
| Smoking habit | 413 (3.18) | 96 (3.63) | 128 (4.2) | 0.025 | 0.054 | 0.03 |
| Statins | 2259 (17.42) | 501 (18.94) | 595 (19.53) | 0.04 | 0.055 | 0.015 |
| Stomach bowel disease | 70 (0.54) | 24 (0.91) | 13 (0.43) | 0.043 | 0.016 | 0.059 |
| Symptomatic slow action drugs for osteoarthritis | 173 (1.33) | 21 (0.79) | 21 (0.69) | 0.053 | 0.064 | 0.012 |
| Thyroid hormone | 802 (6.18) | 188 (7.11) | 199 (6.53) | 0.037 | 0.014 | 0.023 |
| Thyroid nodule | 88 (0.68) | 12 (0.45) | 9 (0.3) | 0.03 | 0.055 | 0.026 |
| Thyroid others | 118 (0.91) | 21 (0.79) | 20 (0.66) | 0.013 | 0.029 | 0.016 |
| Thyroidectomy | 158 (1.22) | 30 (1.13) | 27 (0.89) | 0.008 | 0.033 | 0.025 |
| Tonsillectomy | 178 (1.37) | 37 (1.4) | 29 (0.95) | 0.002 | 0.039 | 0.041 |
| Tuberculosis infection | 84 (0.65) | 13 (0.49) | 11 (0.36) | 0.021 | 0.04 | 0.02 |
| Unspecified arthrosis | 114 (0.88) | 17 (0.64) | 22 (0.72) | 0.027 | 0.018 | 0.01 |
| Urological obstruction | 161 (1.24) | 36 (1.36) | 40 (1.31) | 0.011 | 0.006 | 0.004 |
| Urological surgery | 124 (0.96) | 39 (1.47) | 34 (1.12) | 0.047 | 0.016 | 0.032 |
| Venous insufficiency peripheral | 334 (2.57) | 43 (1.63) | 56 (1.84) | 0.066 | 0.05 | 0.016 |
| Viral hepatitis | 183 (1.41) | 30 (1.13) | 31 (1.02) | 0.025 | 0.036 | 0.011 |
| Vitamins | 88 (0.68) | 28 (1.06) | 31 (1.02) | 0.041 | 0.037 | 0.004 |
| Outpatient readmission | 1,983 (15.29) | 254 (9.6) | 291 (9.55) | 0.173 | 0.175 | 0.002 |
| Sex, women | 9,198 (70.91) | 1,858 (70.25) | 2,098 (68.88) | 0.015 | 0.044 | 0.03 |
| Age at first visit in clinic | 56.3 (44.3-70.4) | 55.7 (44.3-69.4) | 55 (44.9 -69.6) | 0.032 | 0.02 | 0.012 |
| Age at discharge | 56.7 (44.8-70.7) | 56.4 (45.2-70.4) | 56.1 (45.4 – 70.6) | 0.011 | 0.007 | 0.018 |
| Number of previous discharges | 1 (1 – 1) | 1 (1 - 1) | 1 (1 - 1) | 0.006 | 0.041 | 0.037 |
| Number of diagnoses at discharge | 1 (1 – 1) | 1 (1 – 1) | 1 (1 – 1) | 0.005 | 0.001 | 0.004 |
| Number of treatments at discharge | 1 (0 – 2) | 1 (0 – 2) | 1 (0 – 2) | 0.072 | 0.137 | 0.065 |
| Number of visits until discharge, since first visit in clinic | 1 (1 – 2) | 1 (1 – 3) | 1 (1 – 3) | 0.155 | 0.142 | 0.022 |
| Number of visits until discharge, since last discharge | 1 (1 – 1) | 1 (1-2) | 1 (1 - 2) | 0.183 | 0.158 | 0.038 |
| Follow-up days until discharge, per discharge episode | 0 (0 – 0) | 0 (0 – 47) | 0 (0 – 42) | 0.182 | 0.187 | 0.012 |
| Follow-up days until discharge, since last discharge | 0 (0 – 0) | 0 (0 – 0) | 0 (0 – 0) | 0.197 | 0.243 | 0.055 |
| Follow-up days until discharge, since first visit in clinic | 0 (0 – 47) | 0 (0 – 147) | 0 (0 – 160) | 0.285 | 0.329 | 0.057 |
| Follow-up days until discharge, since previous visit in clinic | 0 (0 - 32) | 0 (0 – 56) | 0 (0 - 62) | 0.234 | 0.252 | 0.031 |
| Rosser index at first visit in clinic | 98.6 (98.6 – 99.5) | 98.6 (98.6 – 99.5) | 98.6 (97.3 – 99.5) | 0.028 | 0.004 | 0.037 |
| Rosser index at discharge | 98.6 (98.6 – 99.5) | 98.6 (98.6 – 99.5) | 98.6 (98.6 – 99.5) | 0.013 | 0.014 | 0.001 |
| Rosser index at first episode visit | 98.6 (98.6 – 99.5) | 98.6 (97.3 – 99.5) | 98.6 (97.3 – 99.5) | 0.042 | 0.021 | 0.028 |
| Occupation at first visit in clinic |  |  |  | 0.1 | 0.143 | 0.045 |
| Active | 7,142 (55.06) | 1,551 (58.64) | 1,833 (60.18) |  |  |  |
| Housework | 3,078 (23.73) | 604 (22.84) | 695 (22.82) |  |  |  |
| Retired | 2,468 (19.03) | 417 (15.77) | 433 (14.22) |  |  |  |
| Student | 283 (2.18) | 73 (2.76) | 85 (2.79) |  |  |  |
| Occupation at discharge |  |  |  | 0.115 | 0.129 | 0.028 |
| Active | 7,115 (54.85) | 1,537 (58.11) | 1,809 (59.39) |  |  |  |
| Housework | 3,103 (23.92) | 648 (24.50) | 714 (23.44) |  |  |  |
| Retired | 2,469 (19.03) | 392 (14.82) | 443 (14.54) |  |  |  |
| Student | 284 (2.19) | 68 (2.57) | 80 (2.63) |  |  |  |
| Occupation at first episode visit |  |  |  | 0.112 | 0.132 | 0.031 |
| Active | 7,124 (54.92) | 1,540 (58.22) | 1,818 (59.68) |  |  |  |
| Housework | 3,098 (23.88) | 637 (24.08) | 701 (23.01) |  |  |  |
| Retired | 2,467 (19.02) | 397 (15.01) | 444 (14.58) |  |  |  |
| Student | 282 (2.17) | 71 (2.68) | 83 (2.72) |  |  |  |
| Distress Subscale at first visit in clinic |  |  |  | 0.093 | 0.088 | 0.013 |
| None | 2,390 (18.43) | 429 (16.22) | 497 (16.32) |  |  |  |
| Low | 8,574 (66.10) | 1,725 (65.22) | 1,993 (65.43) |  |  |  |
| Moderate | 1,922 (14.82) | 473 (17.88) | 538 (17.66) |  |  |  |
| High | 85 (0.66) | 18 (0.68) | 18 (0.59) |  |  |  |
| Distress Subscale at discharge |  |  |  | 0.055 | 0.054 | 0.046 |
| None | 2,883 (22.23) | 537 (20.30) | 661 (21.70) |  |  |  |
| Low | 8,413 (64.86) | 1,746 (66.01) | 1,944 (63.82) |  |  |  |
| Moderate | 1,597 (12.31) | 350 (13.23) | 427 (14.02) |  |  |  |
| High | 78 (0.60) | 12 (0.45) | 14 (0.46) |  |  |  |
| Distress at first episode visit |  |  |  | 0.123 | 0.132 | 0.026 |
| None | 2,428 (18.72) | 407 (15.39) | 476 (15.63) |  |  |  |
| Low | 8,552 (65.93) | 1,732 (65.48) | 1,975 (64.84) |  |  |  |
| Moderate | 1,907 (14.70) | 489 (18.49) | 580 (19.04) |  |  |  |
| High | 84 (0.65) | 17 (0.64) | 15 (0.49) |  |  |  |
| Disability Subscale at first visit in clinic |  |  |  | 0.059 | 0.08 | 0.078 |
| None | 5,379 (41.47) | 1,077 (40.72) | 1,171 (38.44) |  |  |  |
| Slight social | 5,243 (40.42) | 1,081 (40.87) | 1,268 (41.63) |  |  |  |
| Severe social and slight physical | 1,402 (10.81) | 279 (10.55) | 365 (11.98) |  |  |  |
| Moderate decrease in mobility | 570 (4.39) | 141 (5.33) | 160 (5.25) |  |  |  |
| Severe decrease in mobility | 286 (2.20) | 46 (1.74) | 65 (2.13) |  |  |  |
| Almost dependent | 86 (0.66) | 19 (0.72) | 17 (0.56) |  |  |  |
| In bed | 5 (0.04) | 2 (0.08) | 0 |  |  |  |
| Disability Subscale at discharge |  |  |  | 0.04 | 0.067 | 0.081 |
| None | 6,038 (46.55) | 1,231 (46.54) | 1,356 (44.52) |  |  |  |
| Slight social | 4,940 (38.08) | 1,032 (39.02) | 1,185 (38.90) |  |  |  |
| Severe social and slight physical | 1,248 (9.62) | 227 (8.58) | 300 (9.85) |  |  |  |
| Moderate decrease in mobility | 437 (3.37) | 87 (3.29) | 132 (4.33) |  |  |  |
| Severe decrease in mobility | 229 (1.77) | 50 (1.89) | 51 (1.67) |  |  |  |
| Almost dependent | 75 (0.58) | 17 (0.64) | 22 (0.72) |  |  |  |
| In bed | 4 (0.03) | 1 (0.04) | 0 |  |  |  |
| Disability Subscale at first episode visit |  |  |  | 0.067 | 0.116 | 0.079 |
| None | 5,442 (41.96) | 1,051 (39.74) | 1,138 (37.36) |  |  |  |
| Slight social | 5,232 (40.34) | 1,088 (41.13) | 1,262 (41.43) |  |  |  |
| Severe social and slight physical | 1,403 (10.82) | 295 (11.15) | 394 (12.93) |  |  |  |
| Moderate decrease in mobility | 532 (4.10) | 136 (5.14) | 162 (5.32) |  |  |  |
| Severe decrease in mobility | 273 (2.10) | 52 (1.97) | 69 (2.27) |  |  |  |
| Almost dependent | 84 (0.65) | 21 (0.79) | 21 (0.69) |  |  |  |
| In bed | 5 (0.04) | 2 (0.08) | 0 |  |  |  |
| (90) Median Rosser Index (w) | 98.6 (98.6 - 99.5) | 98.6 (98.6 - 99.5) | 98.6 (97.3 - 99.5) | 0.036 | 0.023 | 0.016 |
| (90) Median Rosser Index (c) | 98.6 (98.6 - 99.5) | 98.6 (98.6 - 99.5) | 98.6 (98.4 - 99.5) | 0.023 | 0.019 | 0.006 |
| (90) Mean Rosser Index (w) | 98.6 (98.6 - 99.5) | 98.6 (98.6 - 99.5) | 98.6 (97.3 - 99.5) | 0.037 | 0.024 | 0.016 |
| (90) Mean Rosser Index (c) | 98.6 (98.6 - 99.5) | 98.6 (98.6 - 99.5) | 98.6 (98.1 - 99.5) | 0.027 | 0.022 | 0.008 |
| (182) Median Rosser Index (w) | 98.6 (98.6 - 99.5) | 98.6 (98.6 - 99.5) | 98.6 (97.3 - 99.5) | 0.035 | 0.025 | 0.014 |
| (182) Median Rosser Index (c) | 98.6 (98.6 - 99.5) | 98.6 (98.6 - 99.5) | 98.6 (98.0 - 99.5) | 0.023 | 0.021 | 0.005 |
| (182) Mean Rosser Index (w) | 98.6 (98.6 - 99.5) | 98.6 (98.35 - 99.5) | 98.6 (97.3 - 99.5) | 0.036 | 0.026 | 0.014 |
| (182) Mean Rosser Index (c) | 98.6 (98.6 - 99.5) | 98.6 (98.35 - 99.5) | 98.6 (97.9 - 99.5) | 0.026 | 0.022 | 0.007 |
| (90) Median Disability Subscale (w) | 2 (1-2) | 2 (1-2) | 2 (1-2) | 0.035 | 0.08 | 0.044 |
| (90) Median Disability Subscale (c) | 2 (1-2) | 2 (1-2) | 2 (1-2) | 0.018 | 0.066 | 0.047 |
| (90) Mean Disability Subscale (w) | 2 (1-2) | 2 (1-2) | 2 (1-2) | 0.037 | 0.08 | 0.042 |
| (90) Mean Disability Subscale (c) | 2 (1-2) | 2 (1-2) | 2 (1-2) | 0.018 | 0.068 | 0.049 |
| (182) Median Disability Subscale (w) | 2 (1-2) | 2 (1-2) | 2 (1-2) | 0.043 | 0.089 | 0.045 |
| (182) Median Disability Subscale (c) | 2 (1-2) | 2 (1-2) | 2 (1-2) | 0.022 | 0.073 | 0.052 |
| (182) Mean Disability Subscale (w) | 2 (1-2) | 2 (1-2) | 2 (1-2) | 0.045 | 0.087 | 0.042 |
| (182) Mean Disability Subscale (c) | 2 (1-2) | 2 (1-2) | 2 (1-2) | 0.024 | 0.073 | 0.049 |
| (90) Median Distress Subscale (w) | 1 (1 - 1) | 1 (1 - 1) | 1 (1 - 1) | 0.093 | 0.085 | 0.007 |
| (90) Median Distress Subscale (c) | 1 (1 - 1) | 1 (1 - 1) | 1 (1 - 1) | 0.072 | 0.064 | 0.008 |
| (90) Mean Distress Subscale (w) | 1 (1 - 1) | 1 (1 - 1) | 1 (1 - 1) | 0.093 | 0.086 | 0.007 |
| (90) Mean Distress Subscale (c) | 1 (1 - 1) | 1 (1 - 1) | 1 (1 - 1) | 0.073 | 0.064 | 0.008 |
| (182) Median Distress Subscale (w) | 1 (1 - 1) | 1 (1 - 1) | 1 (1 - 1) | 0.097 | 0.097 | 0.001 |
| (182) Median Distress Subscale (c) | 1 (1 - 1) | 1 (1 - 1) | 1 (1 - 1) | 0.075 | 0.07 | 0.004 |
| (182) Mean Distress Subscale (w) | 1 (1 - 1) | 1 (1 - 1) | 1 (1 - 1) | 0.099 | 0.098 | 0.001 |
| (182) Mean Distress Subscale (c) | 1 (1 - 1) | 1 (1 - 1) | 1 (1 - 1) | 0.078 | 0.073 | 0.004 |
|  |  |  |  |  |  |  |

TR: Train dataset; VL: Validation dataset; TS: Test dataset; (90): Mean/median value of all the observations registered in the last 90 day before discharge; (182): Same, but for 182 days; (c): Mean/median value considering the discharge observation; (w): Same, but without considering discharge observation.

**Supplementary Table S12**: Performance measures of the 10 selected outpatient readmission prediction models, and of the reduced final model, based on Precision-Recall, Positive Predictive Value and Negative Predictive Value.

| Model | Precision - Recall | | | | Positive Predictive Value | | | | Negative Predictive Value | | | |
| --- | --- | --- | --- | --- | --- | --- | --- | --- | --- | --- | --- | --- |
|  | TR | V | CV | TS | TR | V | CV | TS | TR | V | CV | TS |
| 1 | 0.452 | 0.165 | 0.403 | 0.177 | 0.327 | 0.166 | 0.301 | 0.161 | 0.909 | 0.931 | 0.910 | 0.928 |
| 2 | 0.451 | 0.175 | 0.408 | 0.186 | 0.325 | 0.176 | 0.306 | 0.168 | 0.905 | 0.931 | 0.910 | 0.927 |
| 3 | 0.457 | 0.171 | 0.409 | 0.181 | 0.335 | 0.171 | 0.306 | 0.170 | 0.909 | 0.932 | 0.912 | 0.931 |
| 4 | 0.459 | 0.168 | 0.406 | 0.180 | 0.332 | 0.175 | 0.305 | 0.162 | 0.909 | 0.933 | 0.911 | 0.929 |
| 5 | 0.457 | 0.170 | 0.409 | 0.179 | 0.335 | 0.175 | 0.304 | 0.166 | 0.909 | 0.932 | 0.912 | 0.930 |
| 6 | 0.449 | 0.183 | 0.398 | 0.177 | 0.331 | 0.186 | 0.310 | 0.174 | 0.904 | 0.933 | 0.911 | 0.930 |
| 7 | 0.450 | 0.183 | 0.402 | 0.178 | 0.336 | 0.178 | 0.307 | 0.173 | 0.906 | 0.931 | 0.908 | 0.928 |
| 8 | 0.455 | 0.177 | 0.412 | 0.180 | 0.334 | 0.180 | 0.308 | 0.167 | 0.905 | 0.932 | 0.909 | 0.926 |
| 9 | 0.455 | 0.177 | 0.412 | 0.179 | 0.334 | 0.178 | 0.308 | 0.168 | 0.905 | 0.932 | 0.909 | 0.927 |
| 10 | 0.455 | 0.177 | 0.412 | 0.180 | 0.332 | 0.178 | 0.307 | 0.170 | 0.906 | 0.932 | 0.910 | 0.927 |
| Reduced final model | - | - | 0.387 | 0.170 | - | - | 0.304 | 0.165 | - | - | 0.907 | 0.924 |
|  |  |  |  |  |  |  |  |  |  |  |  |  |

TR: Results from the Train dataset; CV: Cross-validated results from the Train and Validation datasets; V: Validation dataset; TS: Test dataset.
