# Supplementary figures and images for: Outpatient Readmission in Rheumatology: A Machine Learning Predictive Model of Patient’s Return to the Clinic
Source: J Clin Med. 2019 Aug 2;8(8):1156. doi: 10.3390/jcm8081156 (PMC6723392; doi:10.3390/jcm8081156)

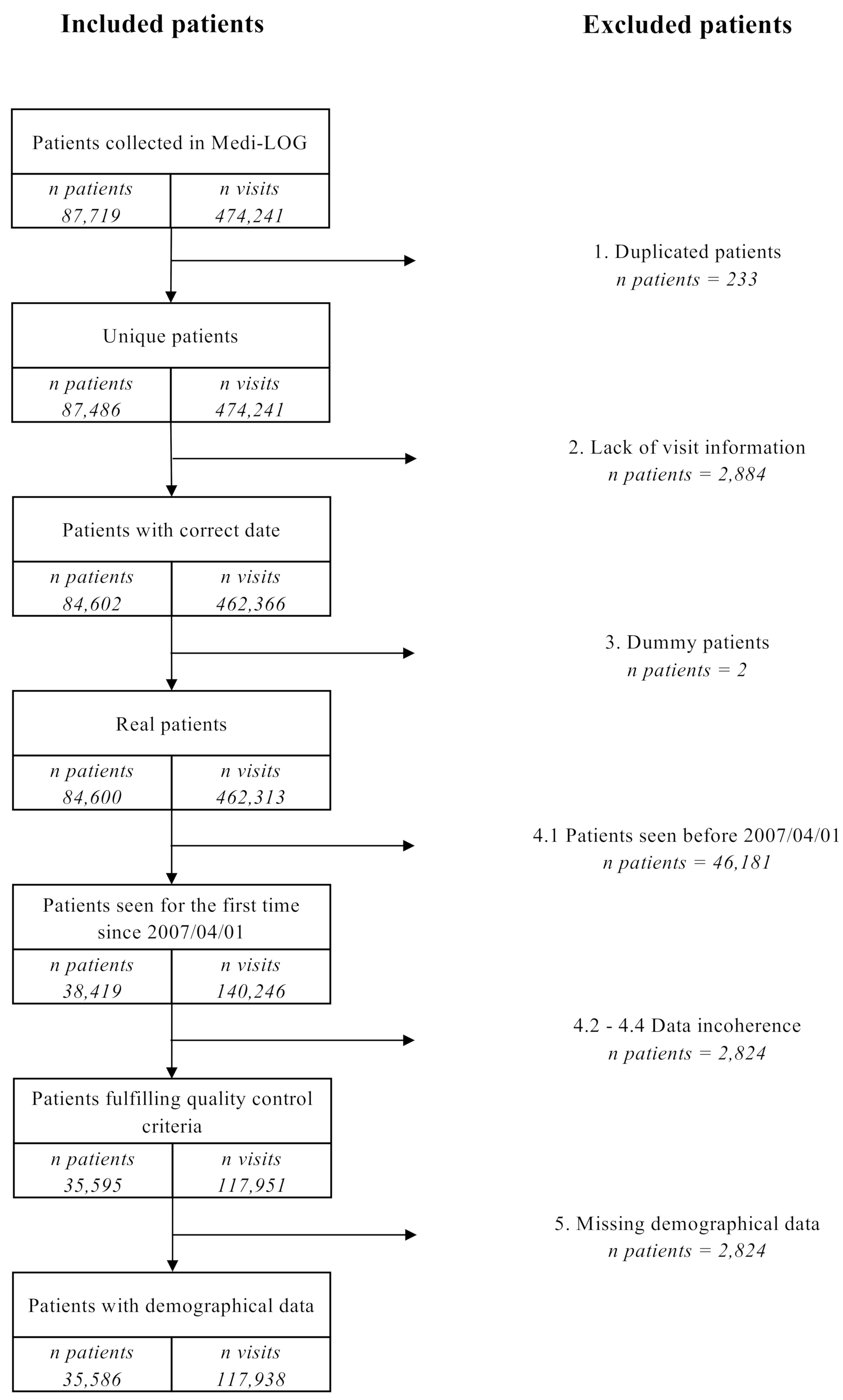

Supplement: Supplementary file 1 [file jcm-08-01156-s001.zip › Supplementary_Figure_S1_R2.tiff]

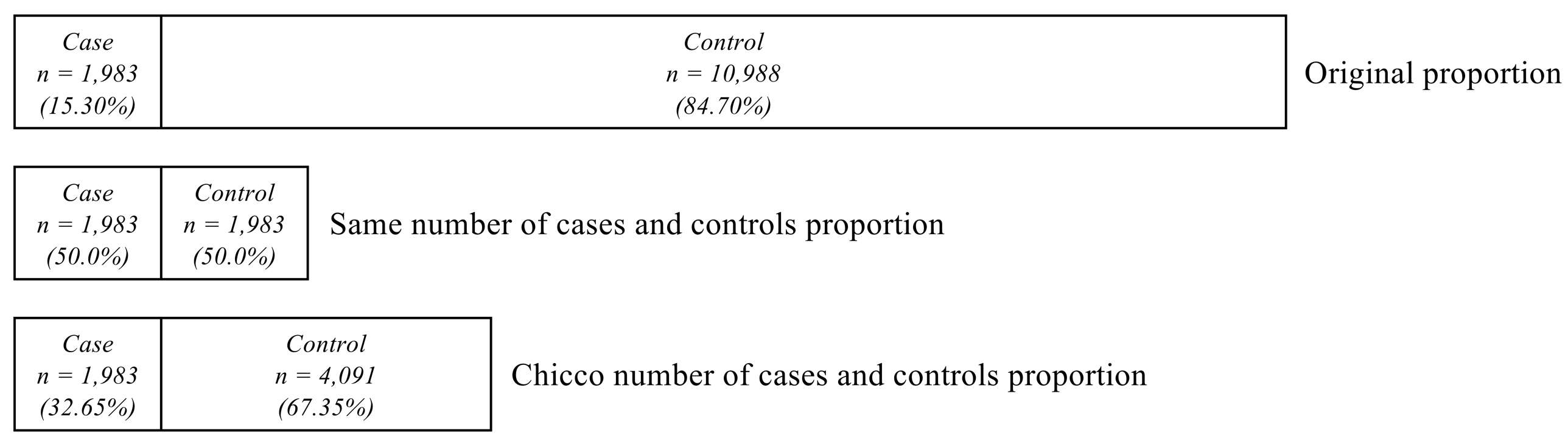

Supplement: Supplementary file 1 [file jcm-08-01156-s001.zip › Supplementary_Figure_S2_R2.tiff]
